# Structural and functional insights into *S*-thiolation of human serum albumins

**DOI:** 10.1038/s41598-018-19610-9

**Published:** 2018-01-17

**Authors:** Fumie Nakashima, Takahiro Shibata, Kohei Kamiya, Jun Yoshitake, Ryosuke Kikuchi, Tadashi Matsushita, Isao Ishii, Juan A. Giménez-Bastida, Claus Schneider, Koji Uchida

**Affiliations:** 1 Graduate School of Bioagricultural Sciences, Nagoya University, Nagoya, 464-8601 Japan; 2 PRESTO, Japan Science and Technology Agency (JST), Kawaguchi, Saitama, 332-0012 Japan; 3 Institute for Innovation for Future Society, Nagoya University, Nagoya, 464-8601 Japan; 4 Department of Medical Technique, Nagoya University Hospital, Nagoya, 466-8560 Japan; 5 Department of Clinical Laboratory and Blood Transfusion, Nagoya University Hospital, Nagoya, 466-8560 Japan; 6 Department of Health Chemistry, Showa Pharmaceutical University, Tokyo, 194-8543 Japan; 7 Department of Pharmacology and Vanderbilt Institute of Chemical Biology, Vanderbilt University Medical School, Nashville, Tennessee 37232 USA; 8 Graduate School of Agricultural and Life Sciences, The University of Tokyo, Tokyo, 113-8657 Japan

## Abstract

Human serum albumin (HSA) is the most abundant serum protein, contributing to the maintenance of redox balance in the extracellular fluids. One single free cysteine residue at position 34 is believed to be a target of oxidation. However, the molecular details and functions of oxidized HSAs remain obscure. Here we analyzed serum samples from normal subjects and hyperlipidemia patients and observed an enhanced *S*-thiolation of HSA in the hyperlipidemia patients as compared to the control individuals. Both cysteine and homocysteine were identified as the low molecular weight thiols bound to the HSAs. Intriguingly, *S*-thiolations were observed not only at Cys34, but also at multiple cysteine residues in the disulfide bonds of HSA. When the serum albumins from genetically modified mice that exhibit high levels of total homocysteine in serum were analyzed, we observed an enhanced *S*-homocysteinylation at multiple cysteine residues. In addition, the cysteine residues in the disulfide bonds were also thiolated in recombinant HSA that had been treated with the disulfide molecules. These findings and the result that *S*-homocysteinylation mediated increased surface hydrophobicity and ligand binding activity of HSA offer new insights into structural and functional alternation of serum albumins via *S*-thiolation.

## Introduction

Post-translational modifications of proteins are one of the most critical biological mechanisms in the dynamic regulations of gene expression, protein stability, activity and localization, and protein-protein interactions. The modifications of proteins are generally catalyzed by specific enzymes, but can also result from non-enzymatic reactions between reactive metabolites and nucleophilic amino acid residues, such as cysteine, one of the critical residues for protein structure and function^1^. Among cysteine modifications, *S*-thiolation is considered to be a reversible, non-enzymatic modification forming mixed disulfides with low molecular weight thiols^2^. The *S*-thiolation reaction proceeds under physiological as well as oxidative stress conditions via the reaction of partially oxidized protein sulfhydryls (sulfenic acid or thiyl radical intermediates) with low molecular weight thiol compounds, such as cysteine or glutathione, or by thiol/disulfide exchange reactions^3–5^. This modification can not only be regarded as a protective mechanism against the terminal or irreversible oxidation of cysteine residues but also be involved in the regulation of the function and activity of proteins.

HSA is the most abundant serum protein, contributing to the maintenance of colloid osmotic blood pressure and the transport of a variety of endogenous and exogenous compounds, including fatty acids, amino acids, bilirubin, hormones and drugs throughout the body^6^. HSA contains 35 cysteine residues, 34 of which form disulfide bridges and only one free sulfhydryl group exists, located at position 34^7–10^. One of the most significant characteristics of the molecular structure of HSA is the presence of a reactive free sulfhydryl group at Cys34. Under physiological conditions, this residue is regarded as the target of oxidation and exists as reduced and oxidized forms. Oxidized HSA is present as a mixed disulfide with cysteine or glutathione, or as an oxyacid, such as in sulfinic acid, sulfonic acid or a similar derivative^11,12^. It has been shown that the ratio of reduced/oxidized form of HSA is related to age and pathological conditions^13–17^. In healthy adults, about 70–80% of Cys34 in albumin exists in the free sulfhydryl form, the rest as a disulfide with thiol compounds^18^. The redox conversion of HSA has been considered to be mainly involved in the maintenance of redox balance, and the value for the reduced HSA fraction on HSA might reflect the redox buffering capacity in the body^14^. However, the molecular details for the formation of oxidized HSAs and their new function associated with altered circulating redox balance remains obscure. In the present study, based on an apparent high abundance of the *S*-thiolated HSAs with low molecular weight thiols, such as cysteine and homocysteine, in the hyperlipidemia patients, we establish that the *S*-thiolation takes place not only at the single free thiol group (Cys34) but also at multiple cysteine residues in the disulfide bonds of HSA. In addition, we show that *S*-homocysteinylation mediates increased surface hydrophobicity and ligand binding activity of HSA. These data not only offer new insights into *S*-thiolation of HSA, but also provide a possible link connecting impaired serum redox balance and structural and functional alternation of serum albumins.

## Results

### Presence of an abnormal serum albumin associated with hyperlipidemia

**Figure 1:**
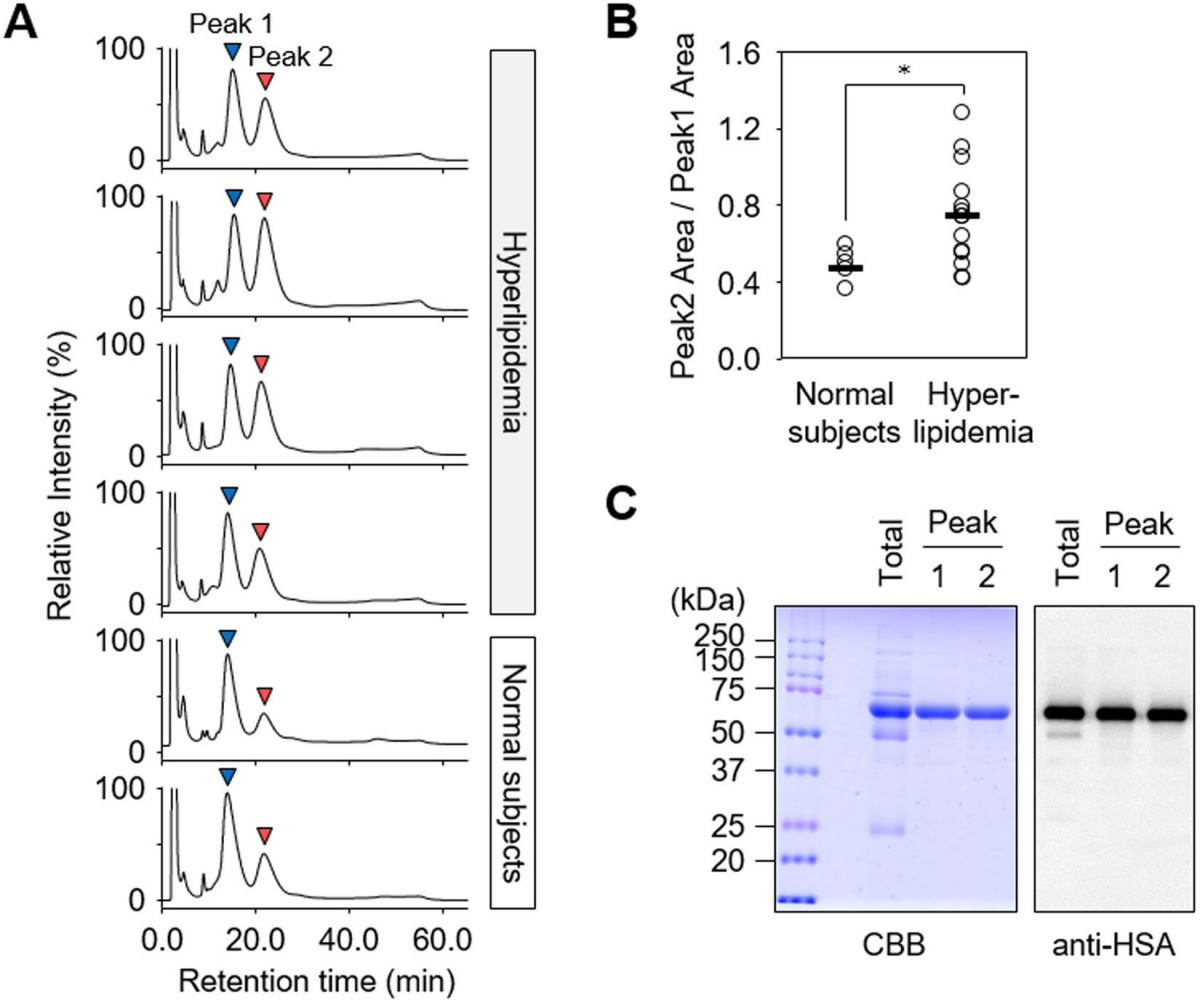
Analysis of serum protein from hyperlipidemia patients. (**A**) Representative chromatograms of anion-exchange liquid chromatography of human serum. Sera from normal subjects and hyperlipidemia patients were submitted for HPLC using an anion-exchange column. (**B**) Comparing the ratio of peak 2 to peak 1 between normal subjects and hyperlipidemia patients. The ratio of peak 2 to peak 1 was calculated from the HPLC peak area. Statistical significance was determined by unpaired Student’s *t*-tests comparing normal subjects to Hyperlipidemia sera samples, *p < 0.05. (**C**) Identification of peak 1 and peak 2 protein. Purified peak 1 and peak 2 from human sera were analyzed by SDS-PAGE under reducing conditions followed by Coomassie Brilliant Blue (CBB) staining (*left panel*) and immunoblot analysis using the anti-HSA antibody (*right panel*).

**Figure 2:**
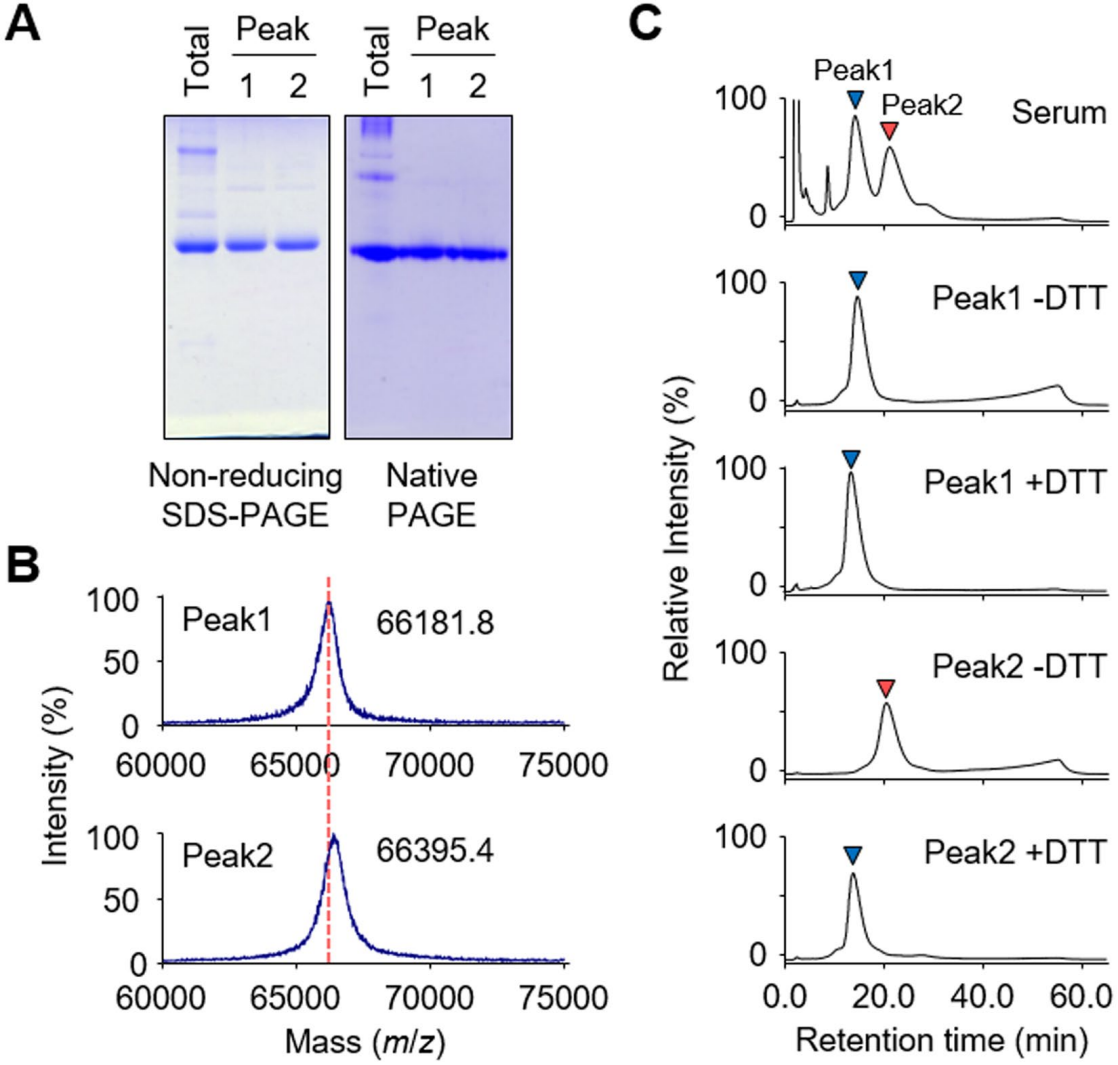
*S*-Thiolation of HSA from hyperlipidemia patients. (**A**) Non-reducing SDS-PAGE (*left panel*) and native-PAGE (*right panel*) analysis of peak 1 and peak 2 HSA. (**B**) Linear mode MALDI-TOF/TOF MS spectrum of peak 1 and peak 2 HSA. (**C**) Anion-exchange liquid chromatography of DTT treated peak 1 and peak 2 HSA. Purified peak 1 and peak 2 HSA were treated with or without DTT for 3 h.

Serum proteins undergo various post-translational modifications, which can be specifically targeted using a combination of chromatographic and mass spectrometry techniques. When we analyzed serum samples from normal subjects (n = 5) and hyperlipidemia patients (n = 15) by HPLC using an anion-exchange column, a remarkable difference in the relative amounts of the major peaks (peaks 1 and 2) was observed (Fig. 1A and Supplemental Fig. 1). The ratio of peak 2 to peak 1 was higher in the hyperlipidemia patients compared to the normal subjects (Fig. 1B). Both peaks contained one major protein, migrating at a molecular mass of ~66 kDa in SDS-PAGE under reducing conditions (Fig. 1C, left panel), which was identical to human serum albumin (HSA) (Supplemental Tables 1 and 2, Supplemental Figs 2 and 3). The result was confirmed by immunoblot analysis using an anti-HSA antibody and by the match in their apparent molecular mass (Fig. 1C, right panel). Non-reducing SDS-PAGE and native PAGE of the two peaks gave protein bands with similar molecular weights (Fig. 2A), suggesting that these two peaks were separated due to binding with low molecular weight compounds.

Depending on the redox state of the free cysteine residue (Cys34), HSA exists in both reduced and oxidized forms^14,19^. In addition, oxidized HSA is a mixture of reversibly and irreversibly oxidized forms. Therefore, it was speculated that HSA gave three peaks because of its redox status. To gain an insight into the redox status of HSA, peak 1 and peak 2 HSAs were analyzed by MALDI-TOF MS. The analysis of peak 1 revealed a peak with m/z 66,181 whereas peak 2 had a peak with m/z 66,395 (Fig. 2B). The peak differs in molecular mass by approximately 200 Da, suggesting the modification of HSA by low molecular weight compounds. DTT treatment resulted in a shift of peak 2 to the same retention time as peak 1, whereas no shift of peak 1 was observed after treatment with the reducing reagent (Fig. 2C), suggesting that peak 1 and peak 2 mainly contain reduced form HSA (redHSA) and oxidized (*S*-thiolated) form HSA (oxHSA), respectively.

### Identification of thiol compounds bound to HSA

**Figure 3:**
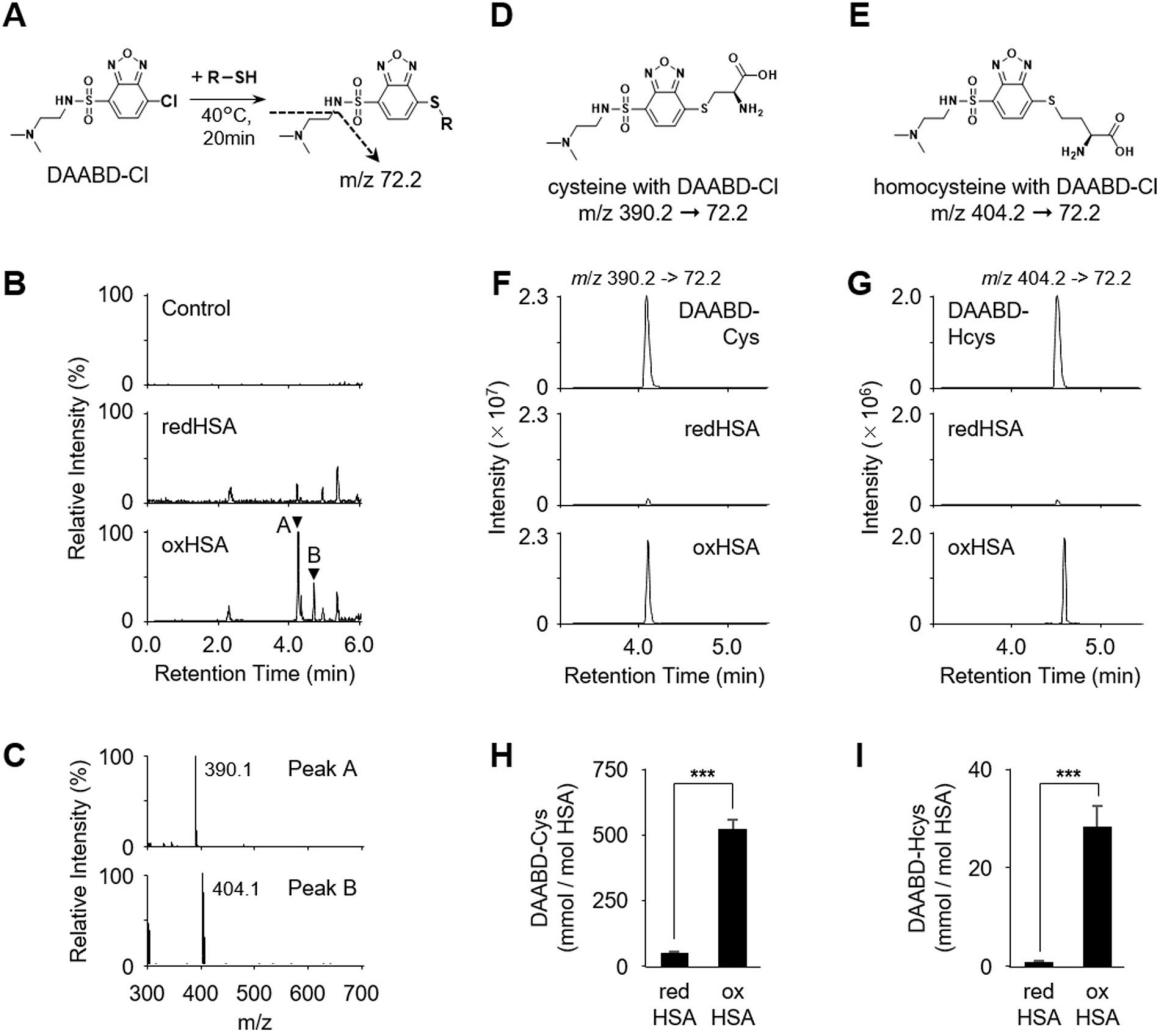
Identification of HSA *S*-cysteinylation and *S*-homocysteinylation. (**A**) The derivatization reaction for thiol compounds using DAABD-Cl. (**B**) LC-MS/MS fragment ion scan of DAABD-Cl derivatized thiol compounds from purified redHSA and oxHSA. Collision-induced dissociation of the [M + H]^+^ of DAABD-Cl derivatized thiol compounds at *m/z* 72.2 at a collision energy of 35 V. (**C**) Positive-ion spectrum of peaks A and B. (**D**) and (**E**) Chemical structure of derivatized thiol compounds. Collision-induced dissociation of the [M + H]^+^ of DAABD-Cl derivatized (**D**) cysteine (390.2 > 72.2) and (**E**) homocysteine (404.2 > 72.2). (**F**) and (**G**), LC-MS/MS analysis in MRM positive ion mode of derivatized cysteine (**F**) and homocysteine (**G**) in the redHSA and the oxHSA from human serum. Authentic derivatives were also analyzed. (**H**) and (**I**) Quantification of HSA-bound cysteine (**H**) and homocysteine (**I**) in redHSA and oxHSA from human sera using LC-MS/MS with the MRM mode. Statistical significance was determined by unpaired Student’s *t*-tests comparing redHSA to oxHSA samples, ***p < 0.005.

To identify the thiol compounds bound to HSA, both redHSA and oxHSA isolated from human sera were treated with tris(2-carboxyethyl)phosphine (TCEP) followed by derivatization with 4-(2-(dimethylamino)ethylaminosulfonyl)-7-chloro-2,1,3-benzoxadiazole (DAABD-Cl)^20,21^ (Fig. 3A). Taking advantage of the fact that DAABD-labeled thiols give a specific fragment ion (*m*/*z* 72.2) in the parent ion scan mode, we performed a comprehensive LC-ESI-MS/MS analysis of the thiol molecules bound to the HSAs. Two peaks, A and B, giving pseudo-molecular ion peaks (M + H)^+^ at *m/z* 390.1 and 404.1, respectively, were uniquely detected in oxHSA (Fig. 3B,C). Based on these parent masses, it was predicted that peaks A and B corresponded to the DAABD derivatives of cysteine and homocysteine, respectively (Fig. 3D,E). Indeed, the product ion spectra of these peaks were identical to those of authentic DAABD-cysteine and DAABD-homocysteine, respectively (Fig. 3F,G). LC-ESI-MS/MS analysis using MRM allowed quantification of cysteine and homocysteine bound to HSA and revealed that both thiols were significantly increased in oxHSA compared to redHSA (Fig. 3H,I). Although the amount of glutathione (GSH) was also increased in oxHSA compared to redHSA, the levels of GSH were much lower than that of cysteine and homocysteine (Supplemental Fig. 4). These data suggest that oxHSA mainly exists as a mixed disulfide with cysteine and/or homocysteine.

### Identification of Cys residues targeted by *S*-thiolation in HSA

**Figure 4:**
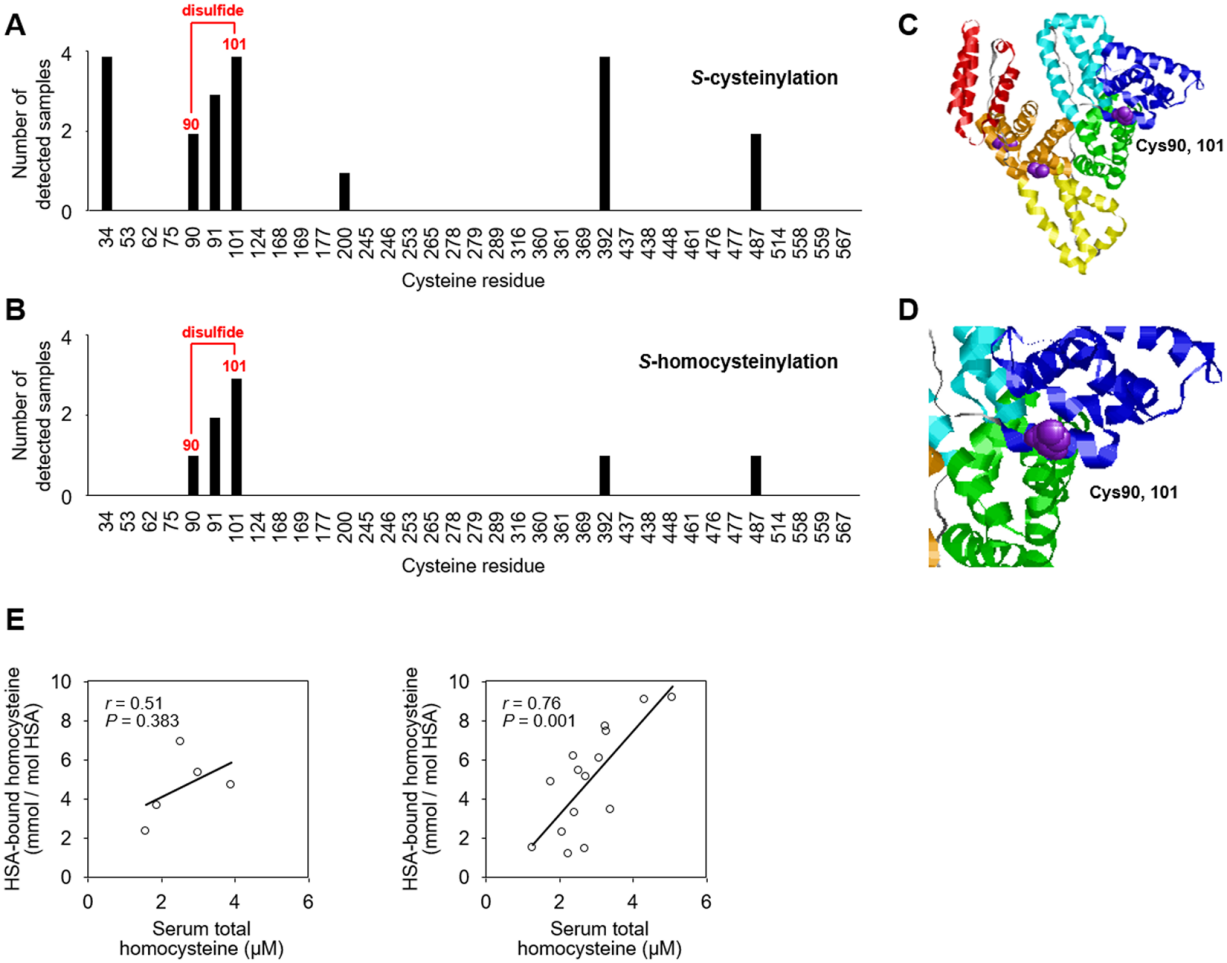
*S*-Thiolation of oxHSA from hyperlipidemia patients. (**A**) and (**B**) Identification of *S*-thiolated cysteine residue of oxHSA from hyperlipidemia patient (n = 4). Number of detected samples of (**A**) *S*-cysteinylation and (**B**) *S*-homocysteinylation in each cysteine residue are shown. (**C**) and (**D**) Rasmol image of three-dimensional structure of HSA. The protein secondary structure is schematically shown and the domains are colored-coded as follows: IA, blue; IB, sky blue; IIA, green; IIB, yellow; IIIA, orange; IIIB, red. Cys90 and Cys101 are shown in a purple space-filling representation. (**E**) Graph illustrating the relationship between serum total homocysteine and HSA-bound homocysteine in normal subjects (n = 5) (*left panel*) and hyperlipidemia patients (n = 15) (*right panel*).

To characterize the hyperlipidemia-related *S*-thiolation of HSA, oxHSA isolated from hyperlipidemia patients (n = 4) was analyzed by MALDI-TOF/TOF MS. To prevent artefactual formation of disulfide after enzymatic digestion and the release of thiol compounds, HSA samples were subjected to alkylation with iodoacetamide, an irreversible alkylating agent, under non-reducing condition. After digestion with trypsin or V8 protease, the recovered peptides were resolved by reverse-phase nano-LC and analyzed using MALDI-TOF/TOF MS. *S*-Thiolation of HSA is expected to occur on Cys34, the only cysteine with a free sulfhydryl group. However, in all 4 patient samples, *S*-cysteinylation was detected not only at Cys34 but also at other cysteine residues, Cys101 and Cys392, that form an intramolecular disulfide bond in native HSA. We also observed *S*-cysteinylation at Cys91 in 3 patient samples, at Cys90 and Cys487 in 2 patient samples, and at Cys200 in 1 patient sample (Fig. 4A, Supplemental Table 3 and Supplemental Fig. 5). *S*-Homocysteinylation was less prevalent in the patient samples (Fig. 4B, Supplemental Table 4 and Supplemental Fig. 6). In a manner similar to *S*-cysteinylation of HSA, multiple cysteine residues, such as Cys90, and Cys101, appeared to be *S*-homocysteinylated. Since these cysteine residues are located at the drug-binding site (Fig. 4C,D), it was hypothesized that *S*-thiolation might lead to significant structural alterations in the protein. In addition to these *S*-thiolated cysteine residues, multiple cysteine residues, such as Cys53, 265, 316, 438 and 448 were detected as reduced thiols (Supplemental Table 5).

To verify the relationship between the serum levels of low molecular weight thiols and *S*-thiolation of HSA, we quantified the total and HSA-bound thiols, namely cysteine, homocysteine, and GSH, by LC-ESI-MS/MS with MRM mode. Although no significant changes were observed in the serum levels of low molecular weight thiols between hyperlipidemia patients and normal subjects (Supplemental Fig. 7), there was a positive correlation between homocysteine and HSA-bound homocysteine in hyperlipidemia patients (Fig. 4E). No significant correlation between cysteine and HSA-bound cysteine and between GSH and HSA-bound GSH in the sera of normal control and hyperlipidemia subjects was observed (Supplemental Fig. 8).

### *S*-Thiolation of serum albumins in cystathionine β-synthase knockout (CBS KO) and cystathionine γ-lyase knockout (CSE KO) mice

**Figure 5:**
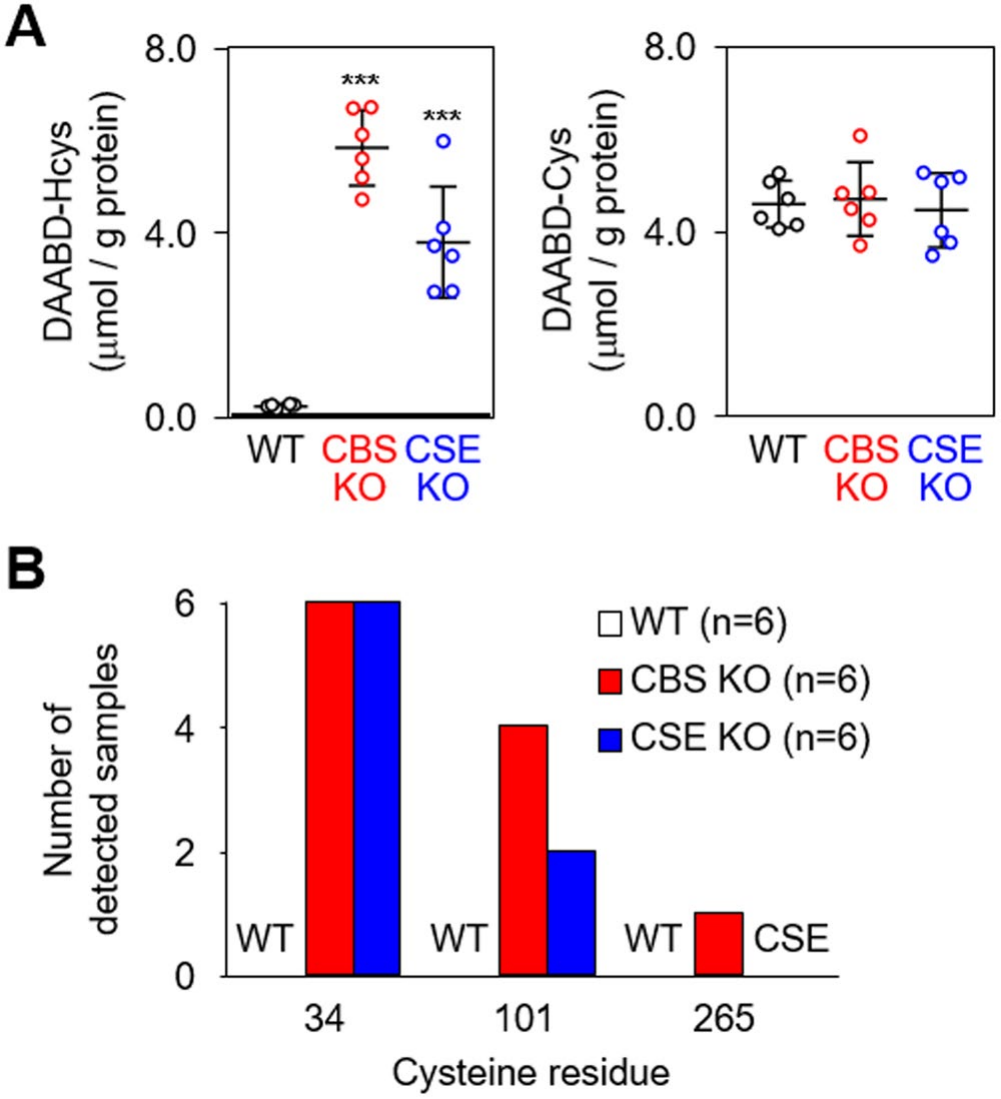
*S*-Thiolation of serum albumin from CBS KO and CSE KO mice. (**A**) Quantification of protein-bound homocysteine (*left*) and cysteine (*right*) in sera from 2-week-old WT, CBS KO and CSE KO mice (all on C57BL/6 J background) using LC-MS/MS. Statistical significance was determined by ANOVA test comparing WT to CBS KO and CSE KO sera samples (n = 6 each), ***P < 0.005. (**B**) Identification of *S*-homocysteinylated cysteine residue of mouse serum albumin from WT, CBS KO and CSE KO mice (n = 6, each). Number of detected samples of *S*-homocysteinylation in each Cys residue is shown.

To further examine the occurrence of *S*-thiolation at cysteine residues in the disulfide bonds of HSA *in vivo*, we utilized two genetic model mice, namely cystathionine β-synthase knockout (CBS KO)^22^ and cystathionine γ-lyase knockout (CSE KO) mice^23^, which exhibit high levels of total homocysteine in serum. Their serum levels of total homocysteine at 2 weeks of age are found to be higher than in age-matched wild-type (WT) mice^23^. Serum samples from WT mice (n = 6), CBS KO mice (n = 6) and CSE KO mice (n = 6) were reduced with TCEP and the resulting free thiol compounds were labeled with DAABD-Cl. As expected, serum proteins were highly *S*-homocysteinylated in both CBS KO and CSE KO mice when compared to WT mice whereas *S*-cysteinylation was unchanged (Fig. 5A). We further analyzed the *S*-homocysteinylation sites in mouse serum albumin using MALDI-TOF/TOF MS. Homocysteine modification of Cys34 was observed in all serum samples (100%) of the CBS KO and CSE KO mice (Fig. 5B). Additional sites of *S*-homocysteinylation were Cys101 and Cys265 in the serum albumins from CBS KO and CSE KO mice, whereas no *S*-homocysteinylation was observed in the sera from WT mice. These modification sites were identical to those of HSA samples from hyperlipidemia patients (Fig. 4).

### *S*-Thiolations of serum albumin by disulfide molecules *in vitro*

**Figure 6:**
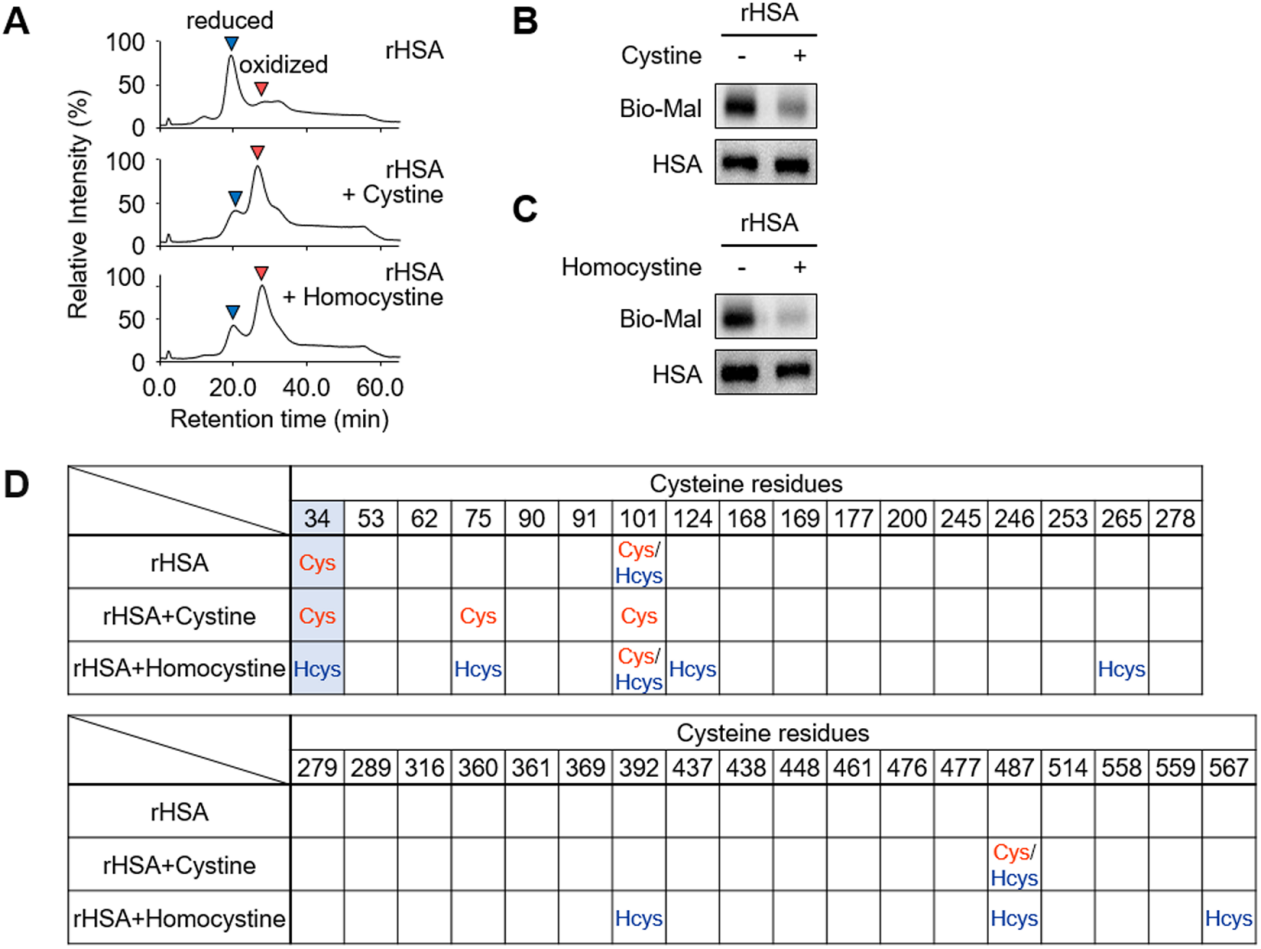
*S*-Thiolation of rHSA by the treatment of cystine and homocystine. rHSA (150 μM) and cystine or homocystine (300 μM) were incubated at 37 °C for 24 h in 0.1 M phosphate buffer (pH 6.7) containing 0.3 M NaCl for *S*-thiolation of HSA. (**A**) HPLC chromatograph of rHSAs. rHSA (*upper*) and cystine (*middle*) or homocystine (*lower*) treated rHSA was analyzed by anion-exchange chromatography by monitoring the excitation at 280 nm and emission at 340 nm. (**B**) and (**C**) Detection of free cysteine residue. rHSA and cystine (**B**) or homocystine (**C**) treated rHSA were labeled with biotin-maleimide and subjected to non-reducing SDS-PAGE followed by immunoblot analysis. (**D**) Identification of *S*-thiolated cysteine residue of cystine or homocystine treated rHSA.

**Figure 7:**
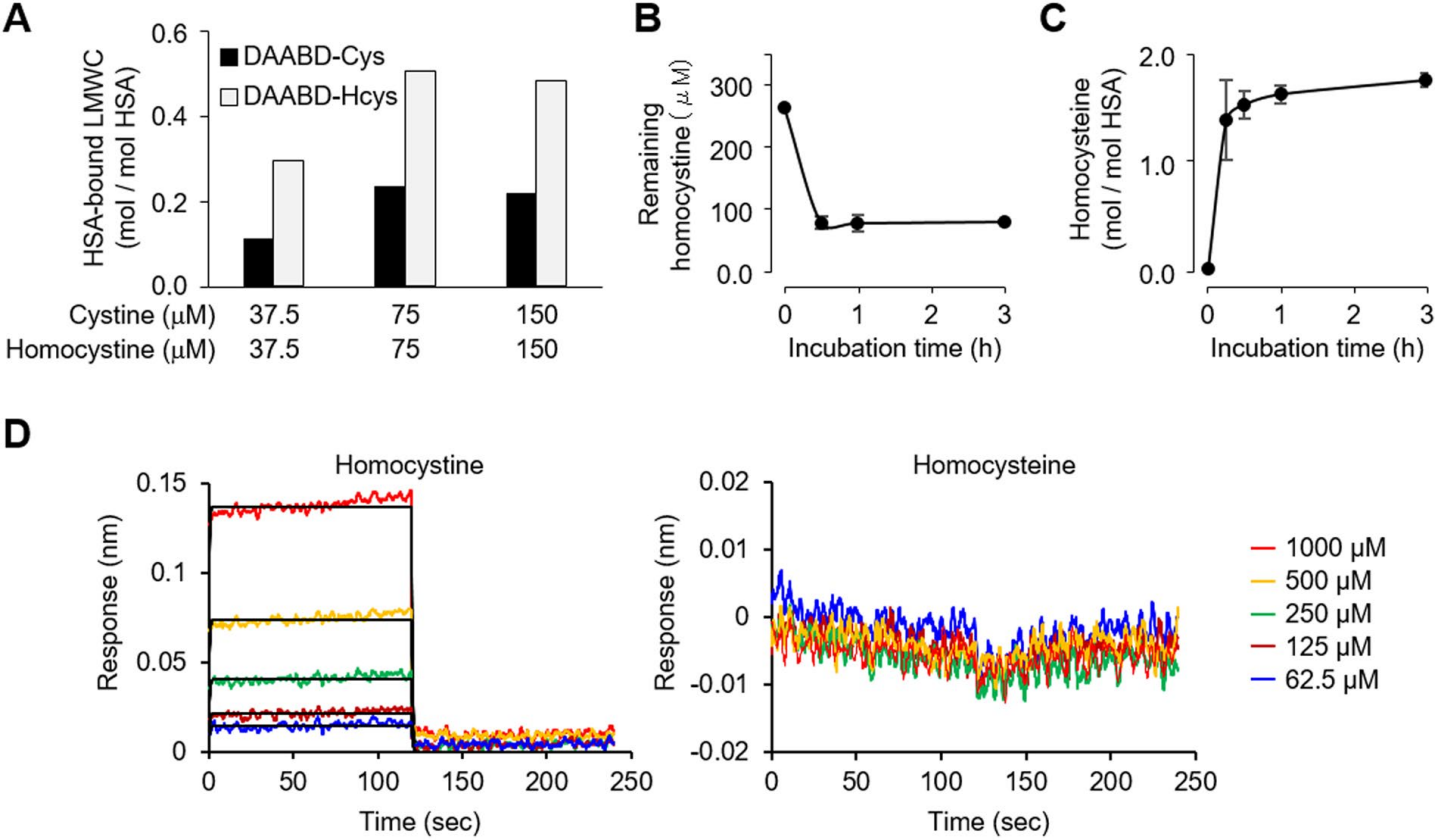
*S*-Homocysteinylation of rHSA. rHSA (150 μM) and same amount of cystine and homocystine (37.5, 75 or 150 μM) were incubated at 37 °C for 24 h in 0.1 M phosphate buffer (pH 6.7) containing 0.3 M NaCl for *S*-thiolation of HSA. (**A**) Quantification of protein-bound cysteine (*black*) and homocysteine (*gray*) in rHSA treated with equal concentrations of cystine and homocystine in the same mixture using LC-MS/MS with MRM mode. (**B**) and (**C**) rHSA (150 μM) and homocystine (300 μM) were incubated at 37 °C for 0–3 h in 0.1 M phosphate buffer (pH 6.7) containing 0.3 M NaCl for *S*-homocysteinylation of HSA. Time-dependent decrease of remaining homocystine in the reaction mixture (**B**) and increase of protein-bound homocysteine (**C**) in a sample which rHSA and homocystine were incubated at 37 °C for 0–3 h. The samples were detected by LC-MS/MS with MRM mode. (**D**) Analysis of interaction between rHSA and homocystine (*left*) or homocysteine (*right*) by the biolayer interferography experiments. The vertical and horizontal axes represent the light shift distance (nm) and asscciation/dissociation time (second), respectively.

The results obtained from hyperlipidemia patients and the CBS KO and CSE KO mice suggest that the disulfide bonds of HSA undergo *S*-thiolation with increasing concentration of the disulfide molecules in serum. Indeed, we observed the *S*-thiolation of cysteine residues in the disulfide bonds in recombinant HSA (rHSA) that had been treated with the disulfide molecules (Fig. 6). Intriguingly, when both cystine and homocystine were present in equal concentrations in the same mixture, the formation of rHSA-bound homocysteine exceeded the formation of rHSA-bound cysteine by about 2.0-fold (Fig. 7A). A time course study revealed that the covalent binding of homocysteine to rHSA increased with time, and during the first 30 min reaction, homocystine immediately reacted with rHSA and formed *S*-homocysteinylated rHSA (Fig. 7B,C). Moreover, interaction between HSA and homocystine or homocysteine was analysed by the biolayer interferography experiments. As shown in Fig. 7D, homocystine can significantly interact with HSA, whereas homocysteine cannot. This result was associated with the observation that homocystine, but not homocysteine, induces the oxidation of HSA (Supplemental Fig. 9).

### Changes in the ligand binding potentials of HSA via *S*-homocysteinylation

**Figure 8:**
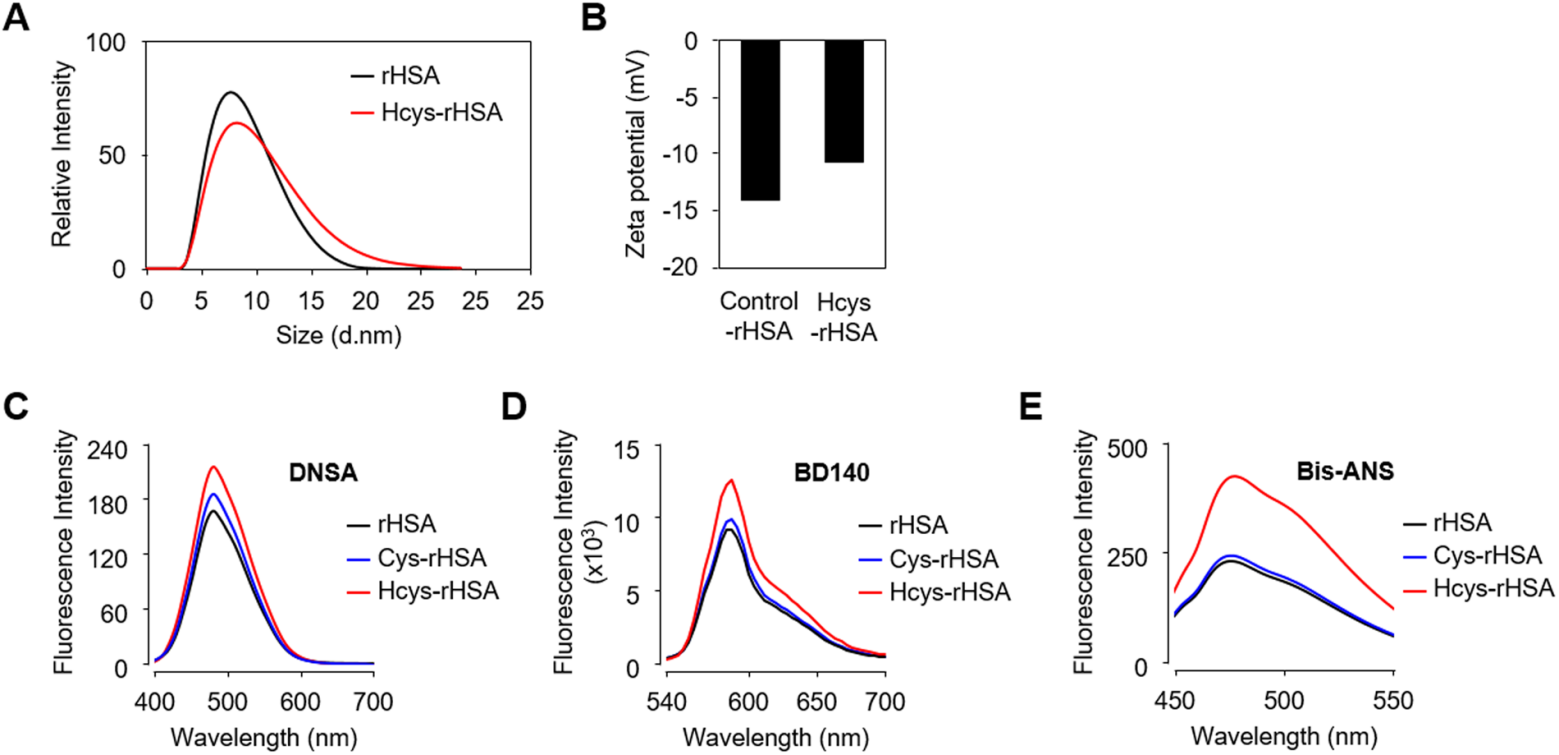
Structural changes of HSA by *S*-thiolation. (**A**) and (**B**) Effects of *S*-homocysteinylation on particle size distribution (**A**) and Zeta potential (**B**) of HSA molecules. (**C**) and (**D**) Analysis of binding ability of modified rHSAs using multiplex fluorescent probes. DNSA for site I (**C**) and BD-140 for site II (**D**). Fluorescence with excitation at 365 nm was measured. (**E**) Analysis of hydrophobic region of modified rHSAs surface using the hydrophobicity probe bis-ANS. Fluorescence with excitation at 394 nm was measured.

We finally examined the effect of *S*-homocysteinylation on the structural properties of HSA. To this end, the particle size distribution was measured by dynamic light scattering analysis. The particle size of HSA was increased by *S*-homocysteinylation (Fig. 8A). We also evaluated the zeta potential, a surface electrical characteristic, of Hcys-rHSA and found the zeta potential of HSA increased to approximately -11 mV upon *S*-homocysteinylation (Fig. 8B). These results suggest the structural changes induced by *S*-homocysteinylation.

HSA displays an extraordinary ligand-binding potential, providing a depot and carrier for many endogenous and exogenous compounds. The ligand binding properties of HSA can largely be explained by two major binding sites, site I and site II^24^, located within specialized cavities in subdomains IIA and IIIA, respectively^25^. Because Cys90 and Cys101 located near site I were identified as the *S*-thiolation sites (Fig. 4), it was expected that *S*-thiolation might impact the ligand-binding capacity of HSA. Indeed, dansylamide (DNSA), a site I specific probe, showed a significant increase in the fluorescence intensity of *S*-homocysteinylated rHSA (Hcys-rHSA) (Fig. 8C). BD-140, a site II specific probe, produced a similar result (Fig. 8D). A more prominent increase in the fluorescence of Hcys-rHSA was observed upon incubation with 4,4′-bis(1-anilino-8-naphthalene sulfonic acid) (bis-ANS), a probe for testing protein surface hydrophobicity (Fig. 8E). These results indicated that *S*-homocysteinylation made the hydrophobic groups of HSA molecules more exposed to the surface.

## Discussion

In the present study, we analyzed serum proteins from normal and hyperlipidemic subjects using anion-exchange liquid chromatography and detected a unique protein peak originating from HSA. We found that this peak was ascribed to the presence of mixed disulfide forms with cysteine and homocysteine (Fig. 3). More strikingly, we established that the *S*-thiolation of HSA occurs not only at Cys34 but also other cysteine residues, such as Cys90 and Cys101, forming an intramolecular disulfide bond in native HSA (Fig. 4). Similar results were also observed in the mouse serum albumin from CBS KO and CSE KO mice (Fig. 5). To the best of our knowledge, this is the first report describing the occurrence of *S*-thiolation at cysteine residues in the disulfide bonds of proteins *in vivo*. Although the mechanism of the thiolation has not yet been experimentally resolved, these findings offer new insights into structural alternation of HSAs under physiological and pathophysiological conditions.

Biological thiols, including homocysteine, play a key role in maintaining an internal redox homeostasis. In human blood, homocysteine is one of the most abundant thiol and its concentration is appropriately10 μM. In plasma, about 60% of the total homocysteine is bound to cysteine residues of protein by disulfide bridges (Hcys-S-S-protein) and most of the remaining 30% circulates in an oxidized form as low molecular weight disulfides, homocystine (Hcys-S-S-Hcys) or homocysteine cysteine mixed disulfide (Hcys-S-S-Cys). Only about 1% of total homocysteine circulates in the free reduced form^26^. Homocysteine like other biological thiols, may play an important role in protection of cells against reactive oxygen species and some reactive intermediate products. However, increased homocysteine levels in blood plasma is a well-known risk factor for cardiovascular diseases such as heart attack^27^ and damage to peripheral veins^28^. In this study, we demonstrated a positive correlation between serum total homocysteine and HSA-bound homocysteine in hyperlipidemia patients, whereas no significant correlations between cysteine and HSA-bound cysteine and between GSH and HSA-bound GSH in the sera of normal control and hyperlipidemia subjects were observed (Fig. 4E and Supplemental Fig. 8). These data and the observation that the serum albumins from CBS KO and CSE KO mice demonstrated an enhanced *S*-homocysteinylation at multiple cysteine residues (Fig. 5) suggest that increased homocysteine levels followed by *S*-homocysteinylation of HSA in blood plasma may directly or indirectly reflect the actual pathogenesis of cardiovascular diseases. Thus, the results of this study may imply the utility and importance of *S*-homocysteinylated HSA as a serum biomarker for human diseases, providing a clue about the possible involvement of homocysteine in human diseases.

Intriguingly, the *in vitro* study revealed that the formation of HSA-bound homocysteine is preferred over formation of HSA-bound cysteine (Fig. 7A). Enthalpy-driven noncovalent interaction accompanying a large entropy penalty has been reported to contribute to thiol-disulfide exchange, leading to the formation of mixed disulfide^29^. It is also known that the protonation of thiolate anion and deprotonation of the buffer contribute to the enthalpy change^29^. Because the pK_α_ of free homocysteine (8.7) is higher than that of free cysteine (8.15), thiolate anion of homocysteine is readily protonated rather than that of cysteine^30^. Therefore, the formation of noncovalent HSA-homocystine complex may be enthalpically more preferred than that of HSA-cystine complex. Thus, the difference in enthalpy, which drives interaction of low molecular weight compounds with proteins, may explain the preferential formation of *S*-homocysteinylated HSA than *S*-cysteinylated HSA. On the other hand, in the experiments using *in vivo* samples, we obtained the unexpected result that all of the hyperlipidemia patient samples showed no *S*-homocysteinylation at Cys34, as opposed to the detection of *S*-cysteinylation at Cys34 in all of these samples (Fig. 4B). This might be associated with our observations that the serum levels of cyst(e)ine (20–100 µM) are much higher than those of homocyst(e)ine (1–5 µM) in hyperlipidemia patients (Fig. 4E and Supplemental Fig. 8A). However, the details for the undetectability of *S*-homocysteinylated Cys34 remain unknown.

HSA contains three domains (I, II and III) each consisting of two subdomains (A and B) with common structural motifs. It is striking to note that most of the *S*-thiolated cysteine residues detected in the serum albumins from the hyperlipidemia patients, CBS KO and CSE KO mice, and in the rHSA treated with homocystine or cystine *in vitro* were located in subdomain A (Figs 4–6). The data and the previous finding that disulfide bond shuffling through thiol/disulfide interchange reactions could take place at cysteine residues in the same subdomain^31^ suggest that the disulfide bonds in subdomain A may be more susceptible to thiol/disulfide interchange reactions than those in other subdomains. Intriguingly, the cysteine residues at positions 90, 91 and 101, which are in the subdomain IA, were identified as highly *S*-thiolated sites in HSA (Figs 4–6). These findings lead us to the hypothesis that subdomain IA may especially be a preferred binding site for cystine and homocystine. Indeed, the serum albumin has been shown to undergo *S*-thiolation with cysteine or homocysteine at Cys34 in subdomain IA^30^. In addition, the binding analysis with the biolayer interferography experiments showed that homocystine, but not homocysteine, can significantly bind to HSA (Fig. 7D). Therefore, it can be speculated that the *S*-thiolation of the disulfide bonds in HSA may be triggered by the thiol/disulfide exchange reactions of Cys34 with disulfide molecules (cystine and homocystine), resulting in the formation of *S*-thiolated Cys34 and low molecular weight thiols (cysteine or homocysteine). These free thiol molecules generated within the subdomain IA may be redox-sensitive and may further cause thiol/disulfide exchange reactions with surrounding disulfide bonds. This chain reaction mechanism may be able to explain the reason for the selective formation of *S*-thiolated cysteines in the subdomain IA and provide new insights into protein *S*-thiolation.

Because disulfide bonds are required for the stability and function of a large number of proteins^32^, modification/disruption of the disulfide bond might change the structure and physiological function of HSA. A previous study has shown that the antioxidant activity of HSA can be inhibited by modification of the disulfide bonds^33^. Using chemical probes to access the hydrophobicity of the protein surface and binding activity, we showed that *S*-homocysteinylation made the hydrophobic groups of HSA molecules more exposed to the surface (Fig. 8). The result is consistent with the observation that *S*-homocysteinylation is preferred over *S*-cysteinylation (Fig. 7A). Homocystine may be more reactive to HSA than cystine because of the presence of chemically inactive methylene group in the homocysteine moiety.

In summary, we identified *S*-thiolated HSAs as a hyperlipidemia-related molecules. Intriguingly, we discovered that *S*-thiolation occurs not only at the single free thiol group, but also at multiple cysteine residues in the disulfide bonds of HSA. Strikingly, *S*-homocysteinylation mediated increased surface hydrophobicity and ligand binding activity of HSA. These results suggest that *S*-homocysteinylation-induced changes in the structure and function of HSA may disturb intercellular and interorgan traffic of endogenous molecules such as fatty acids and hormones, and induce biological responses. Of interest, we have recently observed that *S*-homocysteinylated HSA induces a potent pro-inflammatory response (Nakashima, Shibata, and Uchida, unpublished data). Although further studies are required, our results may provide a new paradigm of the pro-inflammatory effect of *S*-thiolated HSA, which may be exploited in the prevention of and therapy for hyperlipidemia and other chronic inflammatory diseases.

## Methods

### Materials

Goat anti-HSA (ab19180) polyclonal antibody was obtained from Abcam (Cambridge, MA, USA). Bis-ANS (4,4′-Dianilino-1,1′-binaphthyl-5,5′-disulfonic acid, dipotassium salt) was obtained from Thermo Fisher Scientific (San Jose, CA, USA). Recombinant HSA (rHSA) expressed from rice grain (*Oryza sativa*) was obtained from Bio-Verde, Inc. (Kyoto, Japan). 7-chloro-N-[2-(dimethylamino)ethyl]-2,1,3-benzoxadiazole-4-sulfonamide (DAABD-Cl), Dansylamide (DNSA) and BD-140 were obtained from Tokyo Chemical Industry, (Tokyo, Japan).

### Human serum samples

Serum samples were obtained from 5 normal individuals and 15 patients with hyperlipidemia who underwent diagnostic evaluation at the Nagoya University Hospital (Nagoya, Japan). This study was approved by the Ethical Committee of the Nagoya University School of Medicine. Patients were considered to have this lipid disorder based on clinical and biochemical criteria; high total cholesterol [>220 mg/dl], triglyceride [>150 mg/dl], and LDL [>140 mg/dl] levels at the baseline. These samples were originally taken for diagnostic purposes. In this study, we selected the remainder of sera samples from our archive after which they were encoded. In case of research with encoded/anonymous material, informed consent is not required, as long as the researcher is not able to discover the patients identity linked to the research material. When a patient has explicitly refused, it is not allowed to use these sera samples for research. We state that we used encoded sera samples and none of the patients explicitly refused to participate in research.

### Mouse serum samples

Serum samples were obtained from age-matched cystathionine β-synthase knockout (CBS KO) on C57BL/6J background (OMIN 236200)^22^ and cystathionine γ-lyase knockout (CSE KO) mice on C57BL/6J background (OMIN 219500)^23^. This study was approved by the Ethical Committee of the Showa Pharmaceutical University.

### HPLC analysis of human serum albumin

The HPLC-FD system consists of a Model Ternary Gradient Unit LG2080-02, a Model 3-Line Degasser DG-2080-53, and a Model FB-1520S fluorescence detector (excitation wavelength, 280 nm; emission wavelength, 340 nm) together with a Model Intelligent HPLC Pump PU-2080-Plus (JASCO, Tokyo, Japan). A Shodex Asahipak ES-502N 7C column (7.5 mm ID × 100 mm, Showa Denko Co., Tokyo, Japan) was used for the anion-exchange HPLC with an ethanol gradient on 0.05 M sodium acetate, 0.40 M Na_2_SO_4_ (acetate-sulfate buffer), pH 4.85, at the flow-rate of 1.00 mL/min^15,19^. The following ethanol concentrations were used for the analysis: 0–1 min, 0%; 1–50 min, linear increase from 0 to 10%; 50–55 min, linear decrease from 10 to 0%; 55–60 min, 0%. All solvents were filtered through a filter unit (0.22 μm, pore size, TPP) and the samples were filtered through filter units (Millex-GV 0.22 μm Filter Unit, Millipore, Bedford, MA, USA) before use.

### Isolation of human serum albumin from sera

Human serum albumin was isolated using ITSIPREP™ Albumin Segregation Kit-Solvent (ITSI Biosciences, USA). 50 μL of 20 mg/mL sera from normal subjects and hyperlipidemia patients were used for the experiment. Isolated human serum albumin was dissolved in PBS and then protein concentration was determined.

### Immunoblot analysis of serum protein

redHSA and oxHSA isolated from human sera were boiled with Laemmli sample buffer for 10 min at 80 °C. The samples were electrophoresed through a reduced and non-reduced SDS-PAGE (10% polyacrylamide gel) and native-PAGE gel. After electrophoresis, the gel was stained with Coomassie Brilliant Blue or transblotted onto a PVDF membrane and incubated with 0.25% polyvinylpyrrolidone (Sigma, St. Louis, MO, USA) in TBS/T (Tris-buffered saline containing 10% Tween 20) for blocking, washed, and treated with the primary antibodies at 4 °C. After washing with TBS/T, the blots were further incubated for 1 h at room temperature with the HRP-linked secondary anti-IgG antibody. This procedure was followed by the addition of Chemi-Lumi One L western blotting detection reagents (Nacalai Tesque, Tokyo, Japan). The bands were visualized using a WSE-6100LuminoGraph I (ATTO, Tokyo, Japan).

### MALDI-TOF/TOF MS analysis for full length protein

The non-digested proteins were directly spotted onto a MALDI target plate with a matrix 4-CHCA. The MALDI plates were analyzed using a Triple TOF 5800 System (AB SCIEX, Foster City, CA, USA). The mass range was set at m/z 50000-70000.

### LC-ESI-MS/MS analysis of thiol compounds from HSA

Mass spectrometric analyses were performed using an ACQUITY Xevo TQD system (Waters, Milford, MA, USA) equipped with an ESI probe and interfaced with a UPLC system (Waters). The sample injection volumes of 7.5 μl each were separated on a Capcell Core ADME column (2.7 µm, 100 × 2.1 mm i.d.; Shiseido Co., Tokyo, Japan) at the flow rate of 0.3 mL/min and 40 °C. A discontinuous gradient of solvent A (H_2_O containing 0.1% formic acid) and solvent B (acetonitrile containing 0.1% formic acid) was used as follows: 0–2 min, 1%; 2–10 min, linear increase from 1 to 50%; 10.1–12 min, 50%; 12.1–15 min, 1%. Mass spectrometric analyses were performed online using ESI-MS/MS in the positive ion mode along with the multiple reaction monitoring mode (cone potential, 30 eV; collision energy, 35 eV). The parent ion scan was as follows: parent of *m/z* 72.2. The MRM mode scan was as follows: DAABD-cysteine; m/z 390.2->72.2, DAABD-homocysteine; 404.2->72.2. The analytical software (MassLynx, version 4.1) was used for the system control and data processing. For the LC-ESI-MS/MS analysis of the thiol compounds, the protein samples were suspended in 100 μL of 25 mM PB (pH 8.0) and reduced by 2.5 mM TCEP for 30 min at 57 °C. Sixty μl of the ultrafiltration fraction under 30 kDa was then suspended in 40 μl of 25 mM PB (pH8.0) and derivatized by 0.5 mM DAABD-Cl. The reaction mixture was incubated at 50 °C for 20 min, and the reaction was stopped with adding a final concentration of 0.05% formic acid.

### MALDI-TOF/TOF MS analysis for S-thiolation of albumin

Isolated oxHSA from hyperlipidemia patient sera or mouse sera (2-week-old) were alkylated by 1.0 mM of iodoacetamide at room temperature for 30 min. Then the sample was proteolyzed with sequence grade modified trypsin (Promega USA, Madison, WI) in 50 mM NH_4_HCO_3_ buffer in the presence of 0.01% Protease MAX surfactant (Promega) for 1 h at 50 °C or sequence grade V8 protease (Promega USA, Madison, WI) in 50 mM PB (pH 7.4) buffer for overnight at 37 °C. The recovered peptides were then resolved by a DiNa Nano-flow LC system (KYA Technologies Corporation, Tokyo, Japan), then directly fractionated onto a MALDI target plate with matrix 4-CHCA. The MALDI plates were analyzed using a Triple TOF 5800 System (AB SCIEX). The mass range was set at m/z 800-4000. Data from MALDI-TOF/TOF MS were converted into a single MASCOT generic format (.mgf) data file and searched using the MASCOT software (Matrix Sciences, London, UK) and run against the Swiss-Prot database. The parameters were: search type, MS/MS ions; enzyme, trypsin or V8 protease; mass values, monoisotropic; number of possible missed cleavages, three; variable modification, carbamidomethylation (Cys), deamidation (Asp and Glu), oxidation (Met), cysteinylation (Cys), and homocysteinylation (Cys); peptide mass tolerance, 50 ppm; fragment mass tolerance, 0.3 Da.

### Preparation of oxidized HSA

*S*-Cysteinylated rHSA (Cys-rHSA) and *S*-homocysteinylated rHSA (Hcys-rHSA) were prepared as previously described^2,34,35^. rHSA (150 μM) and cystine or homocystine (300 μM) were incubated for 24 h in 0.1 M phosphate buffer (pH 6.7), and 0.3 M NaCl at 37 °C. After incubation, the oxidation reaction was stopped cooling followed by extensive dialysis of the solution against cold PBS. In all cases, the proteins were stored at 4 °C until used.

### Structural properties of reduced and oxidized HSA

For the analysis of the binding ability of albumin, HSAs (10 μM) and BD140 (3 μM) or DNSA (10 μM) were incubated in phosphate buffer (pH 7.3) at room temperature, and fluorescence measurements were taken after incubation for 30 min. The compound was excited at 365 nm^36^. The spectroscopic and quantum yield data were measured by a Spark® 10 M spectrophotometer (TECAN, Männedorf, Switzerland). For the analysis of the effective hydrophobicity of albumin, the HSAs (1 μM) was probed with bis-ANS (10 μM) at 25 °C. The compound was excited at 394 nm, and the fluorescence spectra were recorded by a FP-770 fluorometer (Jasco) using 1-cm quartz cells. The zeta potential and particle-size distributions of HSAs (1.0 mg/ml) were measured using a zeta potential analyzer (Zetasizer Nano ZS, Malvern Instruments).

### Biolayer interferography experiments

Homocysteine/homocystine binding to HSA was measured using an Octet RED system (Pall ForteBio Corp., Menlo Park, CA, USA). Samples were dispensed into 96-well microtiter plates at a volume of 200 μL per well. Operating temperature was maintained at 30 °C. To establish a baseline before protein immobilization, Super Streptavidin Biosensors tips (Pall ForteBio) were prewetted with buffer (Pall ForteBio). Then biotinylated rHSA was immobilized onto Super Streptavidin Biosensors. Data were generated automatically by the Octet User Software (version 8.1) and were subsequently analyzed from the text files using Excel 2010. The binding profile of each sample was summarized as an ‘nm shift’ (the wavelength or spectral shift in nanometers), which represented the difference between the start and end of the 5-min sample association step.

### Statistical Analysis

The data represent the mean ± the standard deviation (SD) where indicated. Statistical significance was evaluated by using the unpaired Student’s t test or, when appropriate, ANOVA test.

### Study approval

This study was approved by the Ethical Committee of the Nagoya University School of Medicine. All animal protocols were approved by the animal experiment committee in the Showa Pharmaceutical University. All experimental methods were carried out in accordance with the relevant guidelines and regulations.

## Electronic supplementary material

Supplementary Information
