# Supplementary material for: Structural and functional insights into S-thiolation of human serum albumins
Source: Sci Rep. 2018 Jan 17;8:932. doi: 10.1038/s41598-018-19610-9 (PMC5772555; doi:10.1038/s41598-018-19610-9)
Supplement: Supplementary file 1 — Supplementary Information [file 41598_2018_19610_MOESM1_ESM.pdf]

Structural and functional insights into *S*-thiolation of human serum albumins

Fumie Nakashima[1], *Takahiro Shibata*[1, 2], *Kohei Kamiya*[1], *Jun Yoshitake*[3], *Ryosuke Kikuchi*[4], *Tadashi Matsushita*[5], *Isao Ishii*[6], *Juan A. Giménez-Bastida*[7], *Claus Schneider*[7] *and Koji Uchida*[1, 8, *]

[1]Graduate School of Bioagricultural Sciences, Nagoya University, Nagoya 464-8601, Japan
[2]PRESTO, Japan Science and Technology Agency (JST), Kawaguchi, Saitama 332-0012, Japan
[3]Institute for Innovation for Future Society, Nagoya University, Nagoya 464-8601, Japan
[4]Department of Medical Technique, Nagoya University Hospital, Nagoya 466-8560, Japan.
[5]Department of Clinical Laboratory and Blood Transfusion, Nagoya University Hospital, Nagoya 466-8560, Japan.
[6]Department of Health Chemistry, Showa Pharmaceutical University, Tokyo 194-8543, Japan
[7]Department of Pharmacology and Vanderbilt Institute of Chemical Biology, Vanderbilt University Medical School, Nashville, Tennessee 37232, U.S.A
[8]Graduate School of Agricultural and Life Sciences, The University of Tokyo, Tokyo 113-8657, Japan

Supplemental Information

Table of Contents

Supplemental Method

MALDI-TOF/TOF MS analysis for protein identification.

Supplemental Tables (Table S1-S5)

1. Identification of peak1 protein by MALDI-TOF/TOF MS analysis.
2. Identification of peak2 protein by MALDI-TOF/TOF MS analysis.
3. Masses of *S*-cysteinylated peptides detedcted with MALDI-TOF/TOF MS.
4. Masses of *S*-homocysteinylated peptides detedcted with MALDI-TOF/TOF MS.
5. Masses of cyseine-containing peptides detected with MALDI-TOF/TOF MS.

Supplemental Figures (Figure S1-S9)

1. Analysis of serum protein from hyperlipidemia patients.
2. Identification of peak 1 protein by MALDI-TOF/TOF MS analysis.
3. Identification of peak 2 protein by MALDI-TOF/TOF MS analysis.
4. Quantification of HSA-bound glutathione.
5. Mass spectra of *S*-cysteinylated peptides detedcted with MALDI-TOF/TOF MS.
6. Mass spectra of *S*-homocysteinylated peptides detedcted with MALDI-TOF/TOF MS.
7. Quantification of serum total thiols from normal subjects and hyperlipidemia patients.
8. Relationship between serum total and HSA-bound thiol.
9. HPLC chromatograph of modified rHSAs.

Supplemental Method

MALDI-TOF/TOF MS analysis for protein identification.

After reduction and alkylation of the cysteine residues with acrylamide, protein was separated by sodium dodecyl sulfate−polyacrylamide gel electrophoresis (SDS−PAGE) under reducing conditions. The separated protein band was processed for tryptic digestion using sequence grade modified trypsin (Promega) in 50 mM NH_4HCO_3 buffer overnight at 37 °C. The recovered peptides were then resolved by DiNa Nano-flow LC system (KYA Technologies Corporation), then directly fractionated onto a MALDI target plate with matrix 4-CHCA. The MALDI plates were analyzed using a Triple TOF 5800 System (AB SCIEX), a hybrid triple quadrupole time-of-flight mass spectrometer equipped with an ESI source, and the mass range was set at m/z 800-4000.

Supplemental Tables (Table S1-S5)

Supplemental Table 1. Identification of peak1 protein by MALDI-TOF/TOF MS analysis.

Masses of tryptic peptides of peak 1 detected with MALDI-TOF/TOF MS.

Position	Observed	Mr(expt)	Mr(calc)	Missed Cleavage	Score	Peptide
1 - 10	1149.5907	1148.5834	1148.5686	1	79	DAHKSEVAHR.F
5 - 10	698.3707	697.3634	697.3507	0	31	K.SEVAHR.F
11 - 20	1226.5764	1225.5691	1225.5979	1	66	R.FKDLGEENFK.A
13 - 20	951.423	950.4157	950.4345	0	60	K.DLGEENFK.A
21 - 41	2504.3352	2503.3279	2503.2933	0	108	K.ALVLIAFAQYLQQCPFEDHVK.L + Propionamide (C)
42 - 51	1149.6576	1148.6503	1148.6077	0	85	K.LVNEVTEFAK.T
52 - 64	1526.5875	1525.5802	1525.6024	0	111	K.TCVADESAENCDK.S + 2 Propionamide (C)
65 - 81	1874.9493	1873.942	1874.0084	1	74	K.SLHTLFGDKLCTVATLR.E
65 - 81	1945.9956	1944.9883	1945.0455	1	138	K.SLHTLFGDKLCTVATLR.E + Propionamide (C)
74 - 81	947.5121	946.5048	946.527	0	53	K.LCTVATLR.E + Propionamide (C)
82 - 93	1462.5458	1461.5385	1461.5574	0	102	R.ETYGEMADCCAK.Q + 2 Propionamide (C)
82 - 93	1478.5504	1477.5431	1477.5523	0	110	R.ETYGEMADCCAK.Q + Oxidation (M); 2 Propionamide (C)
94 - 106	1657.7419	1656.7346	1656.7678	1	66	K.QEPERNECFLQHK.D
94 - 106	1728.7906	1727.7833	1727.8049	1	83	K.QEPERNECFLQHK.D + Propionamide (C)
99 - 106	1089.4939	1088.4866	1088.5073	0	43	R.NECFLQHK.D + Propionamide (C)
99 - 114	1939.8612	1938.8539	1938.9006	1	60	R.NECFLQHKDDNPNLPR.L
99 - 114	1940.8407	1939.8334	1939.8846	1	53	R.NECFLQHKDDNPNLPR.L + Deamidated (NQ)
99 - 114	2010.8983	2009.891	2009.9377	1	121	R.NECFLQHKDDNPNLPR.L + Propionamide (C)
99 - 114	2012.0114	2011.0041	2010.9217	1	47	R.NECFLQHKDDNPNLPR.L + Deamidated (NQ); Propionamide (C)
107 - 114	940.44	939.4327	939.441	0	59	K.DDNPNLPR.L
115 - 136	2593.1797	2592.1724	2592.2352	0	55	R.LVRPEVDVMCTAFHDNEETFLK.K
115 - 136	2664.2209	2663.2136	2663.2724	0	71	R.LVRPEVDVMCTAFHDNEETFLK.K + Propionamide (C)
115 - 137	2721.2783	2720.271	2720.3302	1	88	R.LVRPEVDVMCTAFHDNEETFLKK.Y
115 - 137	2792.2959	2791.2886	2791.3673	1	110	R.LVRPEVDVMCTAFHDNEETFLKK.Y + Propionamide (C)
115 - 137	2808.3008	2807.2935	2807.3622	1	59	R.LVRPEVDVMCTAFHDNEETFLKK.Y + Oxidation (M); Propionamide (C)
137 - 144	1055.5667	1054.5594	1054.5811	1	70	K.KYLYEIAR.R
138 - 144	927.475	926.4678	926.4861	0	49	K.YLYEIAR.R
145 - 159	1899.0016	1897.9943	1897.9879	1	79	R.RHPYFYAPELLFFAK.R
146 - 159	1742.9054	1741.8981	1741.8868	0	112	R.HPYFYAPELLFFAK.R
163 - 174	1399.5779	1398.5706	1398.5908	0	78	K.AAFTECCQAADK.A + 2 Propionamide (C)
182 - 186	645.3514	644.3441	644.3493	0	34	K.LDELR.D
182 - 190	1074.535	1073.5277	1073.5353	1	57	K.LDELRDEGK.A
200 - 205	720.3697	719.3624	719.3636	0	37	K.CASLQK.F + Propionamide (C)
213 - 218	673.3677	672.3605	672.3707	0	40	K.AWAVAR.L
234 - 240	789.4512	788.444	788.4644	0	53	K.LVTDLTK.V
241 - 257	2128.8262	2127.8189	2127.8772	0	84	K.VHTECCHGDLLECADDR.A + 3 Propionamide (C)
241 - 262	2556.072	2555.0647	2555.1203	1	26	K.VHTECCHGDLLECADDRADLAK.Y + 2 Propionamide (C)
241 - 262	2627.125	2626.1177	2626.1574	1	63	K.VHTECCHGDLLECADDRADLAK.Y + 3 Propionamide (C)
263 - 274	1386.5608	1385.5535	1385.6133	0	29	K.YICENQDSISSK.L
263 - 274	1386.5917	1385.5844	1385.6133	0	70	K.YICENQDSISSK.L
275 - 286	1574.7875	1573.7802	1573.8207	1	46	K.LKECCEKPLLEK.S + 2 Propionamide (C)
287 - 313	2988.375	2987.3677	2987.3528	0	220	K.SHCIAEVENDEMPADLPSLAADFVESK.D + Propionamide (C)
287 - 313	2989.4785	2988.4712	2988.3368	0	86	K.SHCIAEVENDEMPADLPSLAADFVESK.D + Deamidated (NQ); Propionamide (C)
287 - 313	3004.3772	3003.3699	3003.3477	0	120	K.SHCIAEVENDEMPADLPSLAADFVESK.D + Oxidation (M); Propionamide (C)
318 - 323	695.3401	694.3329	694.3286	0	40	K.NYAEAK.D
318 - 336	2300.1023	2299.095	2299.0983	1	148	K.NYAEAKDVFLGMFLYEYAR.R
324 - 336	1623.8113	1622.804	1622.7803	0	108	K.DVFLGMFLYEYAR.R
324 - 336	1639.7744	1638.7671	1638.7752	0	67	K.DVFLGMFLYEYAR.R + Oxidation (M)
337 - 348	1467.8123	1466.805	1466.8358	1	76	R.RHPDYSVVLLLR.L
338 - 348	1311.7167	1310.7094	1310.7347	0	90	R.HPDYSVVLLLR.L
352 - 359	984.4741	983.4668	983.4811	0	54	K.TYETTLEK.C
360 - 372	1452.5543	1451.547	1451.5632	0	61	K.CCAAADPHECYAK.V + Propionamide (C)
360 - 372	1523.5923	1522.585	1522.6003	0	65	K.CCAAADPHECYAK.V + 2 Propionamide (C)
360 - 372	1594.6331	1593.6258	1593.6374	0	79	K.CCAAADPHECYAK.V + 3 Propionamide (C)
373 - 389	2045.0439	2044.0366	2044.0881	0	131	K.VFDEFKPLVEEPQNLIK.Q
390 - 402	1600.6633	1599.656	1599.7239	0	48	K.QNCELFEQLGEYK.F
390 - 402	1671.7059	1670.6986	1670.761	0	97	K.QNCELFEQLGEYK.F + Propionamide (C)
390 - 410	2542.2466	2541.2393	2541.2686	1	125	K.QNCELFEQLGEYKFQNALLVR.Y
390 - 410	2613.2869	2612.2796	2612.3057	1	149	K.QNCELFEQLGEYKFQNALLVR.Y + Propionamide (C)
390 - 410	2614.4141	2613.4068	2613.2897	1	110	K.QNCELFEQLGEYKFQNALLVR.Y + Deamidated (NQ); Propionamide (C)
403 - 410	960.5303	959.523	959.5552	0	63	K.FQNALLVR.Y
414 - 428	1639.91	1638.9027	1638.9305	1	122	K.KVPQVSTPTLVEVSR.N
415 - 428	1511.7891	1510.7818	1510.8355	0	128	K.VPQVSTPTLVEVSR.N
415 - 428	1511.9017	1510.8944	1510.8355	0	106	K.VPQVSTPTLVEVSR.N
437 - 444	986.4459	985.4386	985.4473	1	43	K.CCKHPEAK.R + Propionamide (C)
445 - 466	2702.3389	2701.3316	2701.339	1	65	K.RMPCAEDYLSVVLNQLCVLHEK.T + 2 Propionamide (C)
467 - 475	1002.5547	1001.5474	1001.5506	1	27	K.TPVSDRVTK.C
476 - 484	1095.4795	1094.4722	1094.4848	0	51	K.CCTESLVNR.R + Propionamide (C)
476 - 484	1166.5199	1165.5126	1165.522	0	59	K.CCTESLVNR.R + 2 Propionamide (C)
485 - 500	1853.8702	1852.8629	1852.9029	0	68	R.RPCFSALEVDETYVPK.E
485 - 500	1924.8923	1923.885	1923.94	0	86	R.RPCFSALEVDETYVPK.E + Propionamide (C)
501 - 519	2273.9648	2272.9575	2273.031	0	136	K.EFNAETFTFHADICTLSEK.E + Propionamide (C)
501 - 519	2275.1055	2274.0982	2274.015	0	28	K.EFNAETFTFHADICTLSEK.E + Deamidated (NQ); Propionamide (C)
501 - 521	2489.1816	2488.1743	2488.1216	1	48	K.EFNAETFTFHADICTLSEKER.Q + Deamidated (NQ)
501 - 521	2559.1133	2558.106	2558.1747	1	177	K.EFNAETFTFHADICTLSEKER.Q + Propionamide (C)
525 - 534	1128.6528	1127.6455	1127.6914	1	94	K.KQTALVELVK.H
526 - 534	1000.5637	999.5564	999.5964	0	71	K.QTALVELVK.H
546 - 557	1342.6814	1341.6741	1341.6275	0	84	K.AVMDDFAAFVEK.C
546 - 557	1358.5752	1357.5679	1357.6224	0	103	K.AVMDDFAAFVEK.C + Oxidation (M)

Supplemental Table 2. Identification of peak2 protein by MALDI-TOF/TOF MS analysis.

Masses of tryptic peptides of peak 2 detected with MALDI-TOF/TOF MS.

Position	Observed	Mr(expt)	Mr(calc)	Missed Cleavage	Score	Peptide
1 – 10	1149.6279	1148.6206	1148.5686	1	82	DAHKSEVAHR.F
5 – 10	698.387	697.3797	697.3507	0	30	K.SEVAHR.F
11 – 20	1226.6522	1225.6449	1225.5979	1	65	R.FKDLGEENFK.A
13 – 20	951.4334	950.4261	950.4345	0	62	K.DLGEENFK.A
21 – 41	2434.2598	2433.2525	2433.2402	0	38	K.ALVLIAFAQYLQQCPFEDHVK.L + Deamidated (NQ)
21 – 41	2504.3069	2503.2996	2503.2933	0	108	K.ALVLIAFAQYLQQCPFEDHVK.L + Propionamide (C)
21 – 41	2505.3181	2504.3108	2504.2773	0	47	K.ALVLIAFAQYLQQCPFEDHVK.L + Deamidated (NQ); Propionamide (C)
42 – 51	1149.5868	1148.5795	1148.6077	0	72	K.LVNEVTEFAK.T
65 – 73	1017.5012	1016.4939	1016.5291	0	57	K.SLHTLFGDK.L
65 – 81	1874.9221	1873.9148	1874.0084	1	88	K.SLHTLFGDKLCTVATLR.E
65 – 81	1945.9714	1944.9641	1945.0455	1	143	K.SLHTLFGDKLCTVATLR.E + Propionamide (C)
74 – 81	947.5287	946.5215	946.527	0	52	K.LCTVATLR.E + Propionamide (C)
82 – 93	1391.4935	1390.4862	1390.5203	0	49	R.ETYGEMADCCAK.Q + Propionamide (C)
82 – 93	1462.5707	1461.5634	1461.5574	0	96	R.ETYGEMADCCAK.Q + 2 Propionamide (C)
82 – 93	1478.6016	1477.5943	1477.5523	0	111	R.ETYGEMADCCAK.Q + Oxidation (M); 2 Propionamide (C)
94 – 106	1657.7701	1656.7628	1656.7678	1	72	K.QEPERNECFLQHK.D
94 – 106	1728.8123	1727.805	1727.8049	1	82	K.QEPERNECFLQHK.D + Propionamide (C)
94 – 106	1729.7687	1728.7614	1728.7889	1	30	K.QEPERNECFLQHK.D + Deamidated (NQ); Propionamide (C)
99 – 106	1089.5018	1088.4945	1088.5073	0	54	R.NECFLQHK.D + Propionamide (C)
99 – 114	1939.8688	1938.8615	1938.9006	1	84	R.NECFLQHKDDNPNLPR.L
99 – 114	1940.8682	1939.8609	1939.8846	1	32	R.NECFLQHKDDNPNLPR.L + Deamidated (NQ)
99 – 114	2010.9178	2009.9105	2009.9377	1	98	R.NECFLQHKDDNPNLPR.L + Propionamide (C)
99 – 114	2012.0138	2011.0065	2010.9217	1	21	R.NECFLQHKDDNPNLPR.L + Deamidated (NQ); Propionamide (C)
107 – 114	940.4733	939.4661	939.441	0	59	K.DDNPNLPR.L
115 – 136	2593.1545	2592.1472	2592.2352	0	32	R.LVRPEVDVMCTAFHDNEETFLK.K
115 – 136	2664.1797	2663.1724	2663.2724	0	77	R.LVRPEVDVMCTAFHDNEETFLK.K + Propionamide (C)
115 – 137	2721.2466	2720.2393	2720.3302	1	67	R.LVRPEVDVMCTAFHDNEETFLKK.Y
115 – 137	2792.2712	2791.2639	2791.3673	1	102	R.LVRPEVDVMCTAFHDNEETFLKK.Y + Propionamide (C)
115 – 137	2808.2727	2807.2654	2807.3622	1	56	R.LVRPEVDVMCTAFHDNEETFLKK.Y + Oxidation (M); Propionamide (C)
137 – 144	1055.636	1054.6287	1054.5811	1	63	K.KYLYEIAR.R
138 – 144	927.4799	926.4726	926.4861	0	44	K.YLYEIAR.R
145 – 159	1898.9993	1897.992	1897.9879	1	78	R.RHPYFYAPELLFFAK.R
146 – 159	1742.8868	1741.8795	1741.8868	0	100	R.HPYFYAPELLFFAK.R
163 – 174	1399.6169	1398.6096	1398.5908	0	64	K.AAFTECCQAADK.A + 2 Propionamide (C)
175 – 181	786.4415	785.4342	785.4469	0	33	K.AACLLPK.L + Propionamide (C)
182 – 190	1074.5723	1073.565	1073.5353	1	57	K.LDELRDEGK.A
200 – 205	720.3903	719.383	719.3636	0	40	K.CASLQK.F + Propionamide (C)
213 – 218	673.3762	672.3689	672.3707	0	40	K.AWAVAR.L
219 – 225	875.5167	874.5094	874.5025	1	33	R.LSQRFPK.A
234 – 240	789.4596	788.4523	788.4644	0	54	K.LVTDLTK.V
241 – 257	2128.8599	2127.8526	2127.8772	0	109	K.VHTECCHGDLLECADDR.A + 3 Propionamide (C)
241 – 262	2556.0796	2555.0723	2555.1203	1	37	K.VHTECCHGDLLECADDRADLAK.Y + 2 Propionamide (C)
241 – 262	2627.1257	2626.1184	2626.1574	1	92	K.VHTECCHGDLLECADDRADLAK.Y + 3 Propionamide (C)
263 – 274	1386.5618	1385.5545	1385.6133	0	59	K.YICENQDSISSK.L
263 – 274	1457.6744	1456.6671	1456.6504	0	56	K.YICENQDSISSK.L + Propionamide (C)
275 – 286	1574.8185	1573.8112	1573.8207	1	72	K.LKECCEKPLLEK.S + 2 Propionamide (C)
287 – 313	2988.3418	2987.3345	2987.3528	0	227	K.SHCIAEVENDEMPADLPSLAADFVESK.D + Propionamide (C)
287 – 313	3004.3215	3003.3142	3003.3477	0	155	K.SHCIAEVENDEMPADLPSLAADFVESK.D + Oxidation (M); Propionamide (C)
318 – 323	695.3604	694.3531	694.3286	0	45	K.NYAEAK.D
318 – 336	2300.1016	2299.0943	2299.0983	1	142	K.NYAEAKDVFLGMFLYEYAR.R
318 – 336	2301.1069	2300.0996	2300.0823	1	82	K.NYAEAKDVFLGMFLYEYAR.R + Deamidated (NQ)
324 – 336	1623.7968	1622.7895	1622.7803	0	100	K.DVFLGMFLYEYAR.R
324 – 336	1639.7778	1638.7705	1638.7752	0	58	K.DVFLGMFLYEYAR.R + Oxidation (M)
337 – 348	1467.783	1466.7757	1466.8358	1	79	R.RHPDYSVVLLLR.L
338 – 348	1311.6967	1310.6894	1310.7347	0	83	R.HPDYSVVLLLR.L
352 – 359	984.4985	983.4912	983.4811	0	73	K.TYETTLEK.C
360 – 372	1523.642	1522.6347	1522.6003	0	48	K.CCAAADPHECYAK.V + 2 Propionamide (C)
360 – 372	1594.6838	1593.6765	1593.6374	0	73	K.CCAAADPHECYAK.V + 3 Propionamide (C)
373 – 389	2045.0261	2044.0188	2044.0881	0	119	K.VFDEFKPLVEEPQNLIK.Q
390 – 402	1600.6648	1599.6575	1599.7239	0	72	K.QNCELFEQLGEYK.F
390 – 402	1671.6873	1670.68	1670.761	0	103	K.QNCELFEQLGEYK.F + Propionamide (C)
390 – 410	2542.2207	2541.2134	2541.2686	1	132	K.QNCELFEQLGEYKFQNALLVR.Y
390 – 410	2613.2551	2612.2478	2612.3057	1	155	K.QNCELFEQLGEYKFQNALLVR.Y + Propionamide (C)
390 – 410	2613.2961	2612.2888	2612.3057	1	54	K.QNCELFEQLGEYKFQNALLVR.Y + Propionamide (C)
390 – 410	2614.2957	2613.2884	2613.2897	1	89	K.QNCELFEQLGEYKFQNALLVR.Y + Deamidated (NQ); Propionamide (C)
403 – 410	960.5396	959.5323	959.5552	0	59	K.FQNALLVR.Y
414 – 428	1639.9016	1638.8943	1638.9305	1	126	K.KVPQVSTPTLVEVSR.N
415 – 428	1511.8065	1510.7992	1510.8355	0	119	K.VPQVSTPTLVEVSR.N
445 – 466	2702.3391	2701.3318	2701.339	1	77	K.RMPCAEDYLSVVLNQLCVLHEK.T + 2 Propionamide (C)
467 – 472	674.3772	673.3699	673.3395	0	43	K.TPVSDR.V
476 – 484	1024.4467	1023.4394	1023.4477	0	48	K.CCTESLVNR.R
476 – 484	1095.4441	1094.4368	1094.4848	0	57	K.CCTESLVNR.R + Propionamide (C)
476 – 484	1166.5387	1165.5314	1165.522	0	62	K.CCTESLVNR.R + 2 Propionamide (C)
485 – 500	1853.8573	1852.85	1852.9029	0	98	R.RPCFSALEVDETYVPK.E
485 – 500	1924.8708	1923.8635	1923.94	0	96	R.RPCFSALEVDETYVPK.E + Propionamide (C)
501 – 519	2202.9299	2201.9226	2201.9939	0	125	K.EFNAETFTFHADICTLSEK.E
501 – 519	2203.9517	2202.9444	2202.9779	0	25	K.EFNAETFTFHADICTLSEK.E + Deamidated (NQ)
501 – 519	2273.9424	2272.9351	2273.031	0	151	K.EFNAETFTFHADICTLSEK.E + Propionamide (C)
501 – 521	2489.0874	2488.0801	2488.1216	1	55	K.EFNAETFTFHADICTLSEKER.Q + Deamidated (NQ)
501 – 521	2489.137	2488.1297	2488.1216	1	73	K.EFNAETFTFHADICTLSEKER.Q + Deamidated (NQ)
501 – 521	2559.0745	2558.0672	2558.1747	1	187	K.EFNAETFTFHADICTLSEKER.Q + Propionamide (C)
525 – 534	1128.6578	1127.6505	1127.6914	1	84	K.KQTALVELVK.H
526 – 534	1000.5624	999.5551	999.5964	0	71	K.QTALVELVK.H
546 – 557	1342.577	1341.5697	1341.6275	0	44	K.AVMDDFAAFVEK.C
546 – 557	1342.6844	1341.6771	1341.6275	0	96	K.AVMDDFAAFVEK.C
546 – 557	1358.6757	1357.6684	1357.6224	0	84	K.AVMDDFAAFVEK.C + Oxidation (M)

Supplemental Table 3. Masses of *S*-cysteinylated peptides detedcted with MALDI-TOF/TOF MS.

Masses of Trypsin digested (**A**) and V8 protease digested (**B**) peptides detected with MALDI-TOF/TOF MS.

A

Position	Observed	Mr (expt)	Mr (calc)	Missed Cleavage	Score	Peptide
21 - 41	2552.2717	2551.2644	2551.2603	0	90	K.ALVLIAFAQYLQQ**C**PFEDHVK.L + Cysteinyl (C34)
94 - 114	2698.1821	2697.1748	2697.2023	2	105	K.QEPERNE**C**FLQHKDDNPNLPR.L + Cysteinyl (C101)
390 - 402	1719.6868	1718.6795	1718.728	0	55	K.QN**C**ELFEQLGEYK.F + Cysteinyl (C392)
485 - 500	1972.8424	1971.8351	1971.907	0	44	R.RP**C**FSALEVDETYVPK.E + Cysteinyl (C487)

B

Position	Observed	Mr (expt)	Mr (calc)	Missed Cleavage	Score	Peptide
18 - 45	3397.6338	3396.6265	3396.6886	2	52	E.NFKALVLIAFA**Q**YLQQ**C**PFEDHVKLVNE.V + Cysteinyl (C34); Deamidated (Q29)
87 - 100	1799.6262	1798.6189	1798.6742	2	44	E.MAD**CC**AKQEPERNE.C + Carbamidomethyl (C90); Cysteinyl (C91)
90 - 100	1544.5686	1543.5613	1543.5523	1	28	D.**CC**AKQEPERNE.C + 2 Cysteinyl (C90, C91)
101 - 119	2410.1028	2409.0955	2409.1682	2	32	E.**C**FLQHKDDNPNLPRLVRPE.V + Cysteinyl (C101)
189 - 208	2256.0796	2255.0723	2255.1514	0	65	E.GKASSAKQRLK**C**ASLQKFGE.R + Cysteinyl (C200)
377 - 393	2148.0085	2147.0012	2147.0391	1	82	E.FKPLVEEPQNLIKQN**C**E.L + Cysteinyl (C392)

Supplemental Table 4. Masses of *S*-homocysteinylated peptides detedcted with MALDI-TOF/TOF MS.

Masses of Trypsin digested (**A**) and V8 protease digested (**B**) peptides detected with MALDI-TOF/TOF MS.

A

Position	Observed	Mr (expt)	Mr (calc)	Missed Cleavage	Score	Peptide
94 - 114	2712.1492	2711.1419	2711.218	2	48	K.QEPERNE**C**FLQHKDDNPNLPR.L + HCysteinyl (C101)
485 - 500	1986.8774	1985.8701	1985.9227	0	47	R.RP**C**FSALEVDETYVPK.E + HCysteinyl (C487)

B

Position	Observed	Mr (expt)	Mr (calc)	Missed Cleavage	Score	Peptide
87 - 100	1813.637	1812.6297	1812.6899	2	36	E.MAD**CC**AKQEPERNE.C + Carbamidomethyl (C90); HCysteinyl (C91)
90 - 100	1573.6201	1572.6128	1572.5676	1	12	D.**CC**AK**Q**EPERNE.C + Deamidated (Q94); 2 HCysteinyl (C90, C91)
377 - 393	2161.9646	2160.9573	2161.0547	1	65	E.FKPLVEEPQNLIKQN**C**E.L + HCysteinyl (C392)

Supplemental Table 5. Masses of cysteine-containing peptides detected with MALDI-TOF/TOF MS.

Masses of Trypsin digested (**A**) and V8 protease digested (**B**) peptides detected with MALDI-TOF/TOF MS.

A

Position	Observed	Mr (expt)	Mr (calc)	Missed Cleavage	Score	Peptide
74 – 81	933.4875	932.4802	932.5113	0	47	K.L**C**TVATLR.E + Carbamidomethyl (C75)
94 – 114	2636.1846	2635.1773	2635.2197	2	116	K.QEPERNE**C**FLQHKDDNPNLPR.L + Carbamidomethyl (C101)
198 – 205	890.4734	889.4661	889.5055	1	55	R.LKCASLQK.F
263 – 274	1443.5825	1442.5752	1442.6347	0	76	K.YI**C**ENQDSISSK.L + Carbamidomethyl (C265)
314 – 323	1140.4976	1139.4903	1139.5281	1	62	K.DVCKNYAEAK.D
390 – 410	2599.2068	2598.1995	2598.29	1	53	K.QN**C**ELFEQLGEYKFQNALLVR.Y + Carbamidomethyl (C392)
485 – 500	1853.8645	1852.8572	1852.9029	0	41	R.RPCFSALEVDETYVPK.E

B

Position	Observed	Mr (expt)	Mr (calc)	Missed Cleavage	Score	Peptide
49 – 60	1327.5275	1326.5202	1326.5762	2	78	E.FAKT**C**VADESAE.N + Carbamidomethyl (C53)
87 – 100	1737.6477	1736.6404	1736.6916	2	82	E.MAD**CC**AKQEPERNE.C + 2 Carbamidomethyl (C90, C91)
101 – 119	2348.1121	2347.1048	2347.1855	2	64	E.**C**FLQHKDDNPNLPRLVRPE.V + Carbamidomethyl (C101)
189 – 208	2194.1082	2193.1009	2193.1688	0	106	E.GKASSAKQRLK**C**ASLQKFGE.R + Carbamidomethyl (C200)
312 – 321	1156.475	1155.4677	1155.523	1	74	E.SKDVCKNYAE.A
312 – 321	1213.491	1212.4837	1212.5445	1	65	E.SKDV**C**KNYAE.A + Carbamidomethyl (C316)
359 – 368	1101.4072	1100.3999	1100.4379	0	36	E.K**C**CAAADPHE.C + Carbamidomethyl (C361)
369 – 376	974.3856	973.3783	973.4215	1	47	E.CYAKVFDE.F
369 – 376	1031.4116	1030.4043	1030.443	1	51	E.**C**YAKVFDE.F + Carbamidomethyl (C369)
377 – 393	2028.95	2027.9427	2028.035	1	67	E.FKPLVEEPQNLIKQNCE.L
377 – 393	2085.9751	2084.9678	2085.0564	1	106	E.FKPLVEEPQNLIKQN**C**E.L + Carbamidomethyl (C392)
426 – 442	1955.9292	1954.9219	1954.9829	0	73	E.VSRNLGKVGSK**CC**KHPE.A + 2 Carbamidomethyl (C438, C448)
480 – 495	1891.8563	1890.849	1890.9258	2	29	E.SLVNRRP**C**FSALEVDE.T + Carbamidomethyl (C487)

Supplemental Figures (Figure S1-S9)

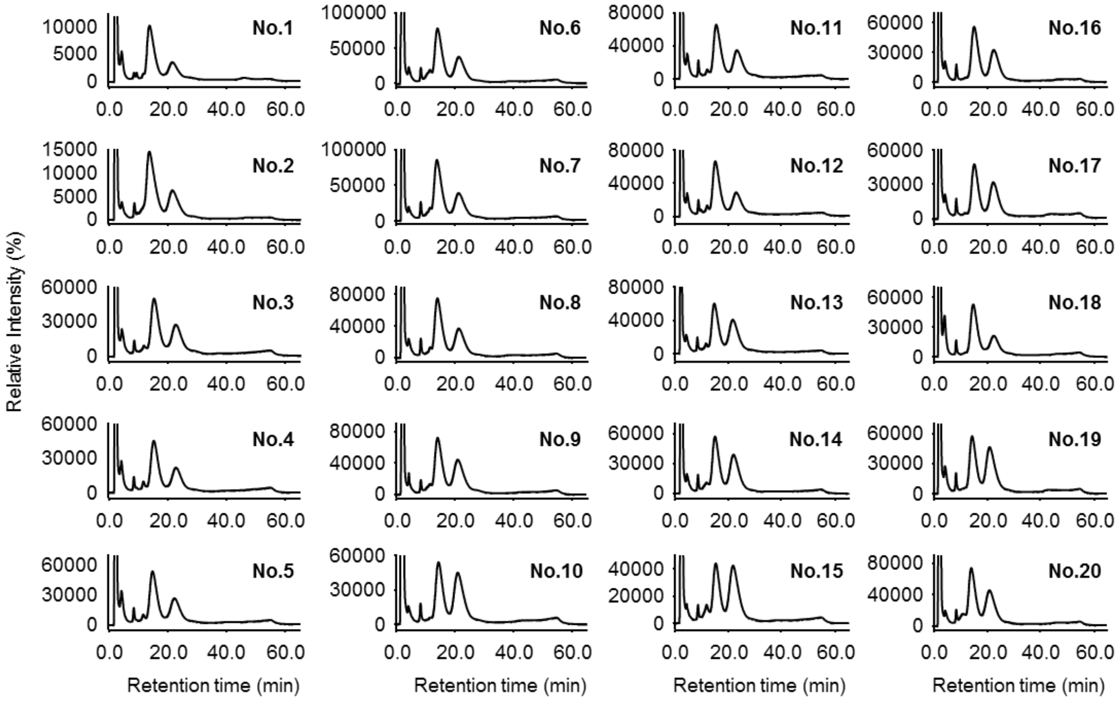

Supplemental Figure 1. Analysis of serum protein from hyperlipidemia patients. Anion-exchange liquid chromatography of human serum. Serum from healthy subjects (No.1-5) and hyperlipidemia patients (No. 6-20) were submitted to HPLC using an anion-exchange column.

1	DAHKSEVAHR	FKDLGEENFK	ALVLIAFAQY	LQQCPFEDHV	KLVNEVTEFA
51	KTCVADESAE	NCDKSLHTLF	GDKLCTVATL	RETYGEMADC	CAKQEPERNE
101	CFLQHKDDNP	NLPRLVRPEV	DVMCTAFHDN	EETFLKKYLY	EIARRHPYFY
151	APELLFFAKR	YKAAFTECCQ	AADKAACLLP	KLDELRDEGK	ASSAKQRLKC
201	ASLQKFGERA	FKAWAVARLS	QRFPKAEFAE	VSKLVTDLTK	VHTECCHGDL
251	LECADDRADL	AKYICENQDS	ISSKLKECCE	KPLLEKSHCI	AEVENDEMPA
301	DLPSLAADFV	ESKDVCKNYA	EAKDVFLGMF	LYEYARRHPD	YSVVLLLRLA
351	KTYETTLEKC	CAAADPHECY	AKVFDEFKPL	VEEPQNLIKQ	NCELFEQLGE
401	YKFQNALLVR	YTKKVPQVST	PTLVEVSRNL	GKVGSKCCKH	PEAKRMPCAE
451	DYLSVVLNQL	CVLHEKTPVS	DRVTKCCTES	LVNRRPCFSA	LEVDETYVPK
501	EFNAETFTFH	ADICTLSEKE	RQIKKQTALV	ELVKHKPKAT	KEQLKAVMDD
551	FAAFVEKCCK	ADDKETCFAE	EGKKLVAASQ	AALGL	

Supplemental Figure 2. Identification of peak 1 protein by MALDI-TOF/TOF MS analysis.

Protein sequence of HSA. Sequence in red is the peptide fragment that was identified in the peak 1.

1	DAHKSEVAHR	FKDLGEENFK	ALVLIAFAQY	LQQCPFEDHV	KLVNEVTEFA
51	KTCVADESAE	NCDKSLHTLF	GDKLCTVATL	RETYGEMADC	CAKQEPERNE
101	CFLQHKDDNP	NLPRLVRPEV	DVMCTAFHDN	EETFLKKYLY	EIARRHPYFY
151	APELLFFAKR	YKAAFTECCQ	AADKAACLLP	KLDELRDEGK	ASSAKQRLKC
201	ASLQKFGERA	FKAWAVARLS	QRFPKAEFAE	VSKLVTDLTK	VHTECCHGDL
251	LECADDRADL	AKYICENQDS	ISSKLKECCE	KPLLEKSHCI	AEVENDEMPA
301	DLPSLAADFV	ESKDVCKNYA	EAKDVFLGMF	LYEYARRHPD	YSVVLLLRLA
351	KTYETTLEKC	CAAADPHECY	AKVFDEFKPL	VEEPQNLIKQ	NCELFEQLGE
401	YKFQNALLVR	YTKKVPQVST	PTLVEVSRNL	GKVGSKCCKH	PEAKRMPCAE
451	DYLSVVLNQL	CVLHEKTPVS	DRVTKCCTES	LVNRRPCFSA	LEVDETYVPK
501	EFNAETFTFH	ADICTLSEKE	RQIKKQTALV	ELVKHKPKAT	KEQLKAVMDD
551	FAAFVEKCCK	ADDKETCFAE	EGKKLVAASQ	AALGL	

Supplemental Figure 3. Identification of peak 2 protein by MALDI-TOF/TOF MS analysis.

Protein sequence of HSA. Sequence in red is the peptide fragment that was identified in the peak 2.

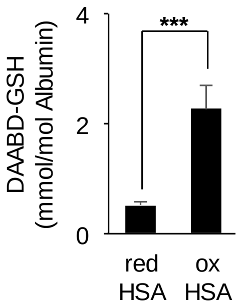

Supplemental Figure 4. Quantification of HSA-bound glutathione.

Protein bound glutathione in redHSA and oxHSA from human sera were quantified using LC-MS/MS with the MRM mode. Statistical significance was determined by unpaired Student's t-tests comparing redHSA to oxHSA samples, ***$p < 0.005$.

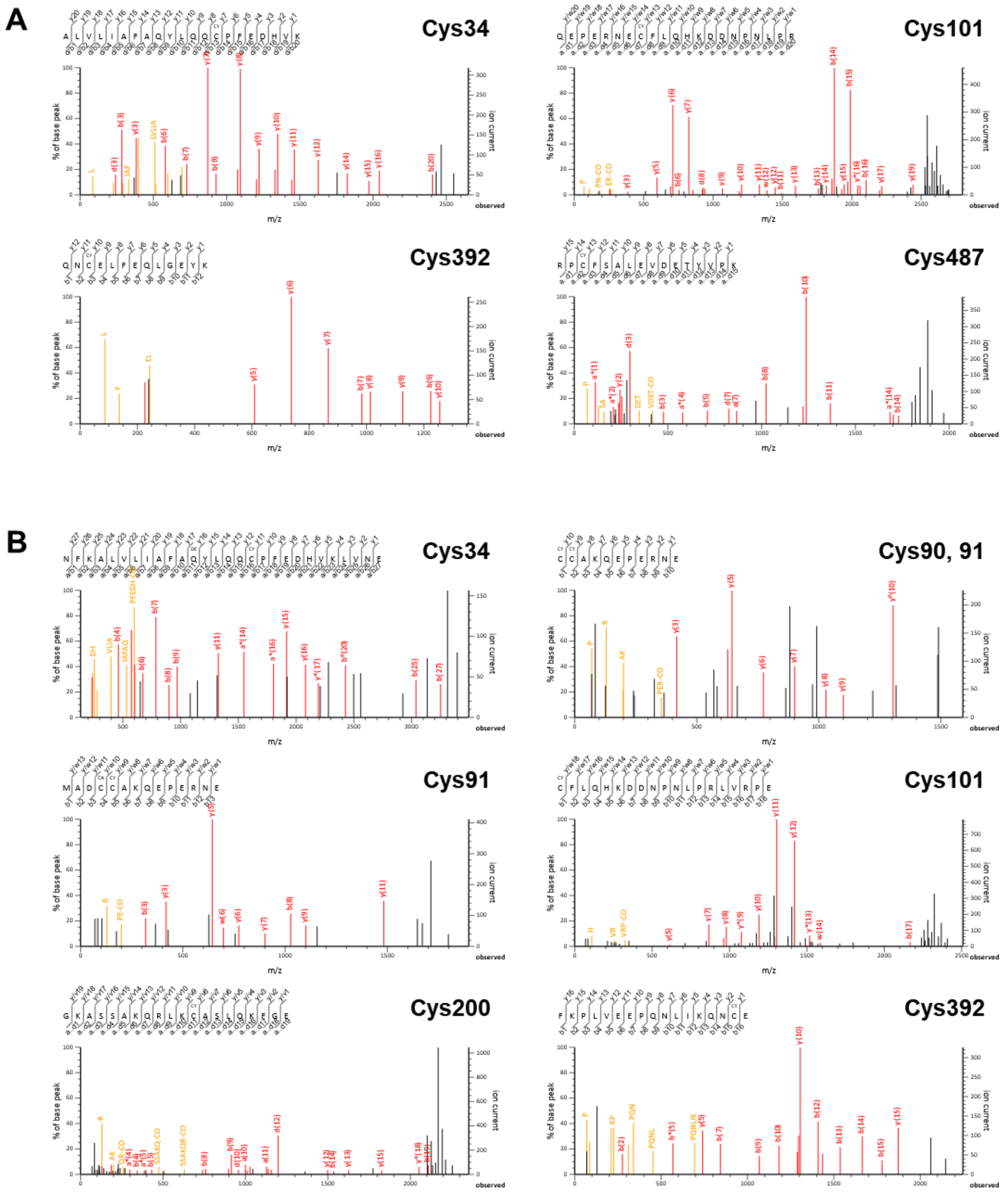

Supplemental Figure 5. Mass spectra of *S*-cysteinylated peptides detedcted with MALDI-TOF/TOF MS.

Mass spectra of Trypsin digested (**A**) and V8 protease digested (**B**) peptides detected with MALDI-TOF/TOF MS. Data represent CA; carbamidomethylation, CY; cysteinylation, and DE; deamidation.

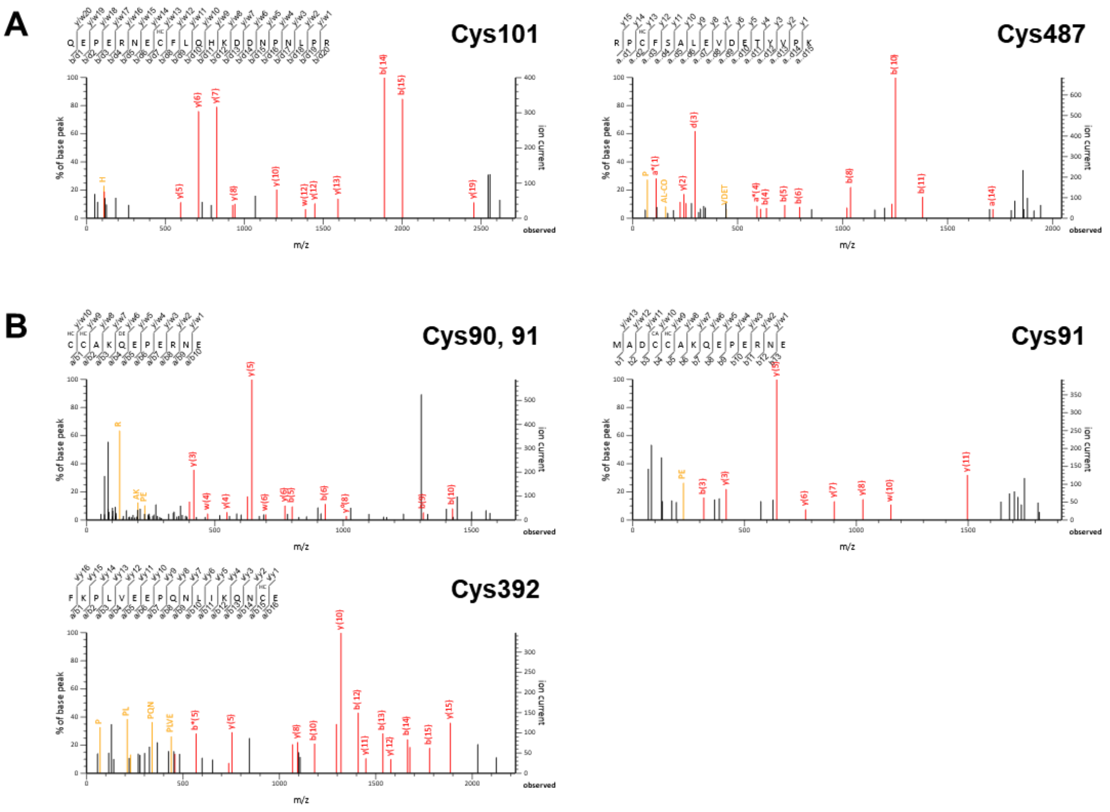

Supplemental Figure 6. Mass spectra of *S*-homocysteinylated peptides detedcted with MALDI-TOF/TOF MS.

Mass spectra of Trypsin digested (**A**) and V8 protease digested (**B**) peptides detected with MALDI-TOF/TOF MS. Data represent CA; carbamidomethylation, HC; homocysteinylation, and DE; deamidation.

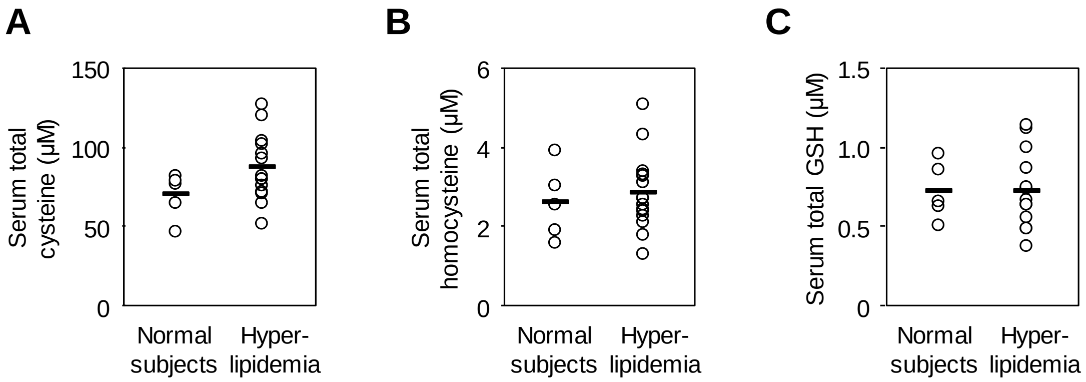

Supplemental Figure 7. Quantification of serum total thiols from normal subject and hyperlipidemia patients.

Quantification of (**A**) cysteine, (**B**) homocysteine, and (**C**) GSH in sera from normal subjects and hyperlipidemia patients were quantified using LC-MS/MS with the MRM mode.

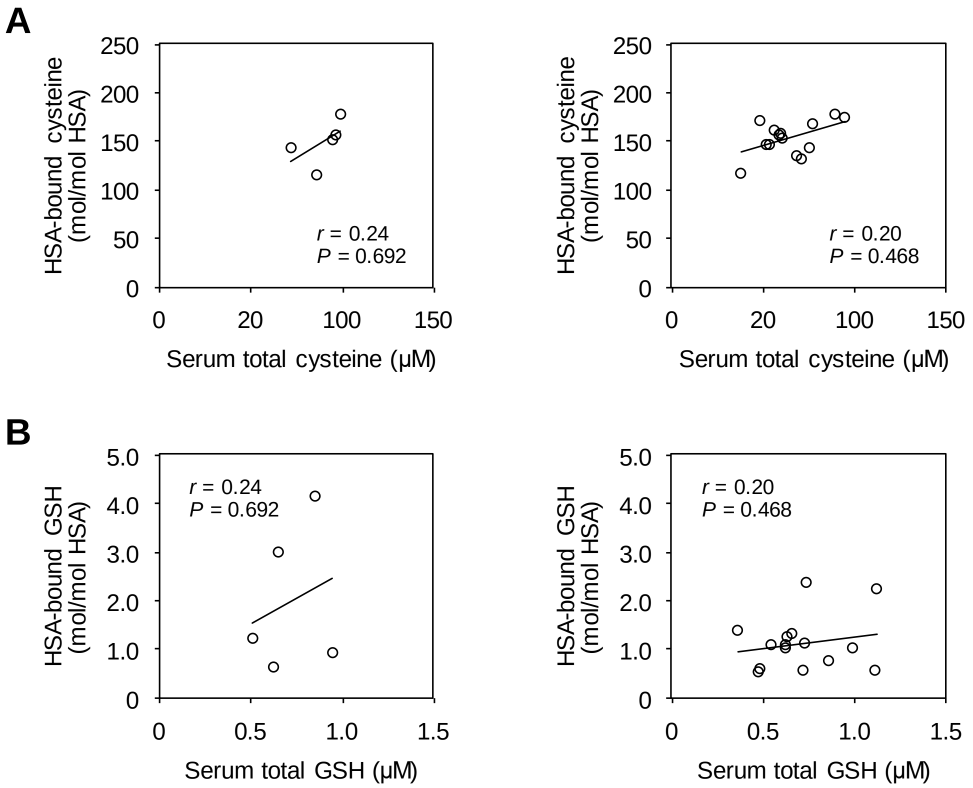

Supplemental Figure 8. Relationship between serum total and HSA-bound thiol. Graph illustrating the relationship between (**A**) serum total cysteine and HSA-bound cysteine in normal subjects (n=5) (*left panel*) and hyperlipidemia patients (n=15) (*right panel*), and (**B**) serum total GSH and HSA-bound GSH in normal subjects (n=5) (*left panel*) and hyperlipidemia patients (n=15) (*right panel*).

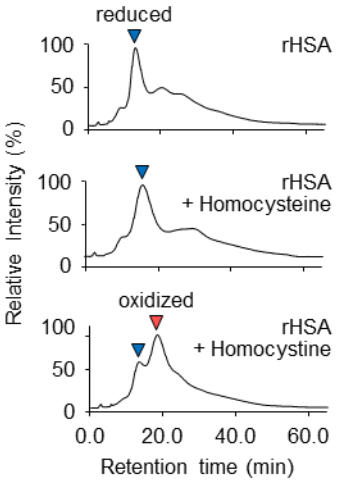

Supplemental Figure 9. HPLC chromatograph of modified rHSAs.

rHSA (150 μM) and homocysteine (600 μM) or homocystine (300 μM) were incubated at 37°C for 24 h in 0.1 M phosphate buffer (pH 6.7) containing 0.3 M NaCl for *S*-thiolation of HSA. rHSA (*top*) and homocysteine (*middle*) or homocystine treated rHSA (*lower*) was analyzed by anion-exchange chromatography by monitoring the excitation at 280 nm and emission at 340 nm.
